# Characterization of Dye-Loaded Poly(lactic-*co*-glycolic acid) Nanoparticles by Comprehensive Two-Dimensional Liquid Chromatography Combining Hydrodynamic and Reversed-Phase Liquid Chromatography

**DOI:** 10.1021/acs.analchem.3c03356

**Published:** 2023-12-13

**Authors:** Joshka Verduin, Luca Tutiš, Alexander J. Becking, Amin Famili, Kelly Zhang, Bob W. J. Pirok, Govert W. Somsen

**Affiliations:** † Department of Chemistry and Pharmaceutical Sciences, Amsterdam Institute of Molecular and Life Sciences (AIMMS), Division of BioAnalytical Chemistry, Vrije Universiteit Amsterdam, De Boelelaan 1085, 1081 HV Amsterdam, The Netherlands; ‡ Centre of Analytical Sciences Amsterdam (CASA), Science Park 904, 1098 XH Amsterdam, The Netherlands; § Synthetic Molecule Pharmaceutical Sciences, Genentech, Inc., 1 DNA Way, South San Francisco, California 94080, United States; ∥ van ’t Hoff Institute for Molecular Sciences (HIMS), Analytical-Chemistry Group, University of Amsterdam, Science Park 904, 1098 XH Amsterdam, The Netherlands

## Abstract

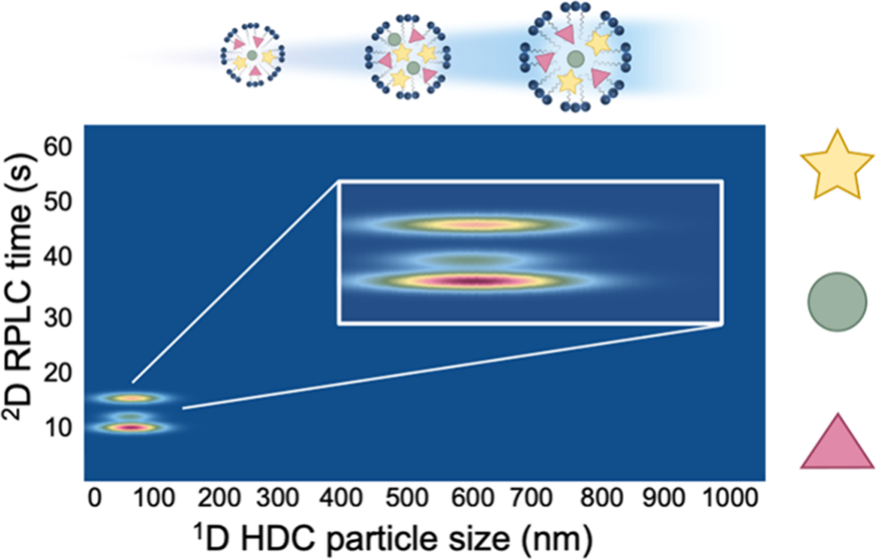

Analytical methods for the assessment of drug-delivery systems (DDSs) are commonly suitable for characterizing individual DDS properties, but do not allow determination of several properties simultaneously. A comprehensive online two-dimensional liquid chromatography (LC × LC) system was developed that is aimed to be capable of characterizing both nanoparticle size and encapsulated cargo over the particle size distribution of a DDS by using one integrated method. Polymeric nanoparticles (NPs) with encapsulated hydrophobic dyes were used as model DDSs. Hydrodynamic chromatography (HDC) was used in the first dimension to separate the intact NPs and to determine the particle size distribution. Fractions from the first dimension were taken comprehensively and disassembled online by the addition of an organic solvent, thereby releasing the encapsulated cargo. Reversed-phase liquid chromatography (RPLC) was used as a second dimension to separate the released dyes. Conditions were optimized to ensure the complete disassembly of the NPs and the dissolution of the dyes during the solvent modulation step. Subsequently, stationary-phase-assisted modulation (SPAM) was applied for trapping and preconcentration of the analytes, thereby minimizing the risk of analyte precipitation or breakthrough. The developed HDC × RPLC method allows for the characterization of encapsulated cargo as a function of intact nanoparticle size and shows potential for the analysis of API stability.

## Introduction

Pharmaceuticals are mostly ingested orally or administered intravenously. However, not all drugs allow for these routes of administration, for instance, when they are too unstable or insoluble in aqueous environments. As a solution, drug-delivery systems (DDSs) can be used. These formulations have first been introduced to the pharmaceutical market decades ago.^1^ DDSs are composed of vehicles that carry an active pharmaceutical ingredient (API). The nanovehicles have several benefits, including administration of nontraditional pharmaceuticals, targeted drug delivery, and regulated release of the medicine.^2,3^

Different carrier types can be employed, such as liposomes, lipid nanoparticles (LNPs), and polymer nanoparticles (NPs). Depending on the carrier, they can encapsulate drugs of different hydrophilicities.^4^ Poly(lactic-*co*-glycolic acid) (PLGA) is a biodegradable polymer that has been used as a carrier in approved pharmaceutical formulations for over 20 years.^5^ Its biocompatibility, stability, tunable chemical properties, and mechanical strength have made PLGA an attractive polymer for drug delivery.^6,7^ PLGA-based DDSs have been applied for the administration of a great variety of medicines.^3,8,9^ Either the PLGA itself or a modified block-*co*-polymer can be used as a carrier. A generic approach to increase the half-life of PLGA nanocarriers is the addition of the more hydrophilic polyethylene glycol (PEG) as a copolymer.^6,10^

For formulation development and quality control of PLGA nanoformulations, multiple properties have to be assessed, such as particle size, release of the API, and stability of the formulation product.^11^ When the size of the NPs is determined, a suitable analytical method must be used that maintains the native state of the nanovehicle. Therefore, techniques that do not require (intensive) sample preparation or harsh solvents that may denature the particles are typically used for determining the particle size. Dynamic light scattering (DLS), differential scanning calorimetry (DSC), and optical or electron microscopy are commonly reported methods.^10,12−14^ These techniques provide information about properties such as the average and distribution of the particle size. Also, separation techniques have proven useful for the analysis of intact NPs, including field-flow fractionation (FFF) and hydrodynamic chromatography (HDC). FFF facilitates the separation of macromolecules from the nano- to micrometer range in an open channel, and the use of mild separation conditions maintains the nanovehicles in their native state. Asymmetrical flow FFF (AF4) has been shown to be a useful technique for DDS analysis.^4,15^ Alternatively, HDC can be used, wherein submicron particles are separated in a packed column to determine the particle size distribution.^16^ The aqueous eluents that are typically used in HDC preserve the native conformation of the nanocarriers.^17,18^ The absence of pores and the corresponding pore stress in HDC allows for more gentle separations than size-exclusion chromatography (SEC).^19^ Furthermore, HDC facilitates separation of particles that would be too large to be separated by SEC.^20^

Besides NP size, polymer length, API content, and chemical integrity are also of interest for drug-loaded polymer NPs. The use of SEC and reversed-phase liquid chromatography (RPLC) have been reported for the determination of the molecular weight of the polymer and loading of the NPs, respectively.^21−24^

A typical workflow to characterize API content involves offline disintegration of the NP formulation followed by the analysis of the API components (and potentially polymer molecules) by RPLC. This allows quantitative determination of API (and possibly polymer) in an aliquot of the sample, but (i) it requires manual sample preparation and (ii) significant information is lost about the relation between nanoparticle size and API loading.

Comprehensive 2D-LC (LC × LC) in principle allows for correlating orthogonal compound properties. In LC × LC, the entire first-dimension (^1^D) effluent is fractionated and subjected to a second-dimension (^2^D) separation.^25,26^ While LC × LC was investigated previously for NP analysis,^18,27^ no studies attempted to simultaneously determine the loading of NPs and their size distribution, as well as the correlation between the two.

In this work, we report a novel HDC × RPLC method for the characterization of the encapsulated compounds as a function of the particle size distribution for dye-loaded PLGA NPs. To our knowledge, this is the first time an HDC method is combined with a non-size-based separation. This is of interest because the direct relation between intact particles and encapsulated compounds thus far has been difficult to determine. Our initial work reported here focuses on the technological development of the 2D-LC method. Model PLGA NPs with encapsulated hydrophobic dyes were used to represent a DDS. Notably, the dye curcumin is a drug candidate that has been formulated in PLGA NPs to enhance drug delivery.^28−34^ Special attention has been paid to the stationary-phase-assisted modulation (SPAM) step to enhance solvent compatibility among the separation dimensions and the analyte sensitivity of the method.

## Experimental Section

### Chemicals and Reagents

Acetone (technical grade) was obtained from VWR Chemicals (Fontenay-sous-Bois, France). Brij L23 nonionic surfactant (30% w/v in water), coumarin-6 (98%), curcumin (from Curcuma longa), formic acid (FA, ≥98%), poly(d,l-lactide-*co*-glycolide)-*block*-poly(ethylene glycol)-*block*-poly(d,l-lactide-*co*-glycolide)-based poly(ester urethane) (PLGA–PEG-PLGA, average *M*_n_ 6000–12,000), Sudan-IV, sodium dodecyl sulfate (SDS, ≥99.0%), sodium azide, and thiourea (≥99.0%) were obtained from Sigma-Aldrich (Darmstadt, Germany). Sodium dihydrogen orthophosphate was obtained from Merck (Darmstadt, Germany). Acetonitrile (ACN, LC-MS grade) was obtained from Biosolve BV (Valkenswaard, The Netherlands). All water used was deionized (Arium 611UV; Satorius, Germany, resistivity 18.2 MΩ cm).

The 3000 series polystyrene (PS) nanosphere standards were obtained from Thermo Scientific (Fremont, CA). The particle diameters were 903 ± 12 nm (P/N: 3900A), 510 ± 7 nm (P/N: 3500A), 401 ± 6 nm (P/N: 3400A), 345 ± 7 nm (P/N: 3350A), 203 ± 4 nm (P/N: 3200A), 100 ± 4 nm (P/N: 3100A), 70 ± 3 nm (P/N: 3070A), and 31 ± 3 nm (P/N: 3030A) (SD and CV values for the standards are reported in Table S1). Green fluorescent Degradex Poly lactic-*co*-glycolic acid (PLGA) dry polymer microspheres and nanospheres were obtained from Phosphorex, Inc. (Hopkinton, MA). The particle diameters reported by the supplier were 468 nm ±239 nm (LGFG500, PLGA NP A) and 189 nm ±34 nm (LGFG200, PLGA NP B).

An HDC mobile phase was prepared by dissolving 6.2 g of sodium dihydrogen orthophosphate, 10.0 g of SDS, 134 mL of Brij L23, and 4.0 g of sodium azide in 866.7 mL water. This stock solution was diluted 20-fold in water for analysis (now referred to as HDC eluent).

**Figure 1:**
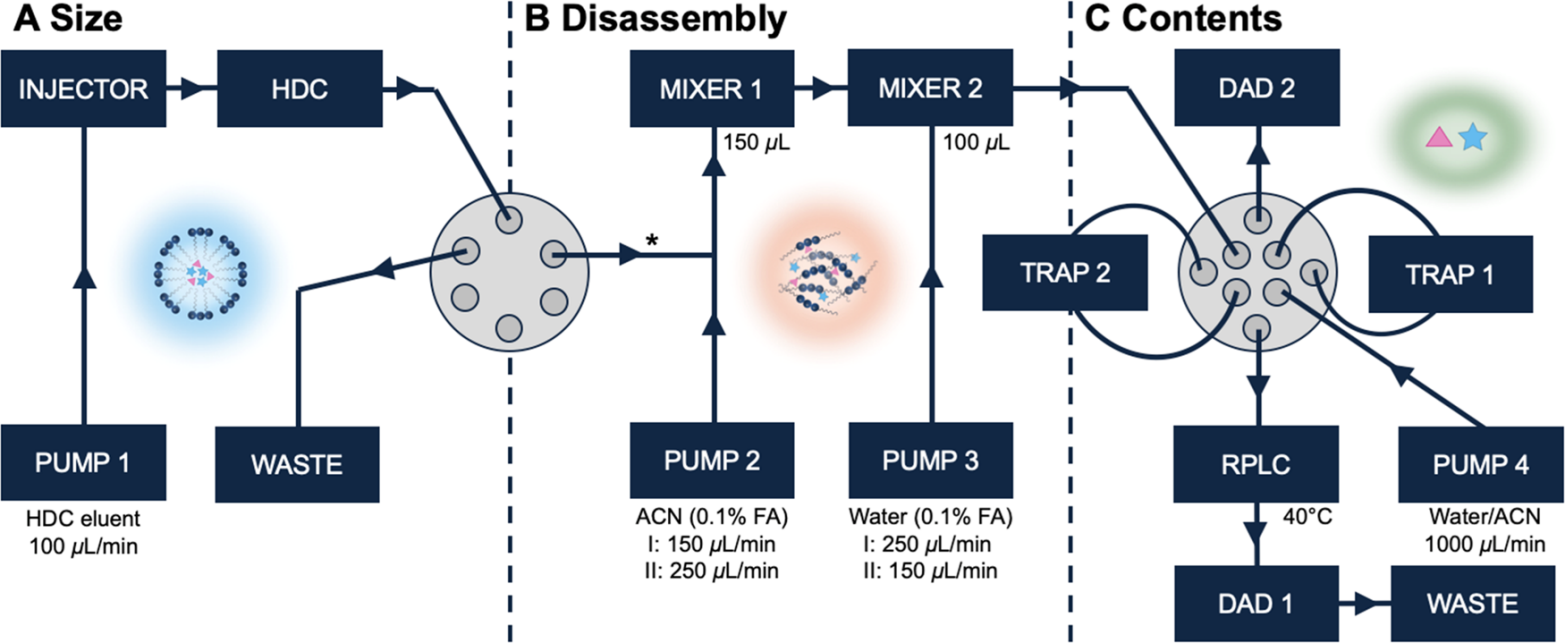
Schematic representation of the HDC × RPLC setup. (A) Intact NP analysis by HDC (^1^D), (B) NP disassembly and dilution, (C) SPAM and RPLC analysis (^2^D) of NP cargo; *, restriction capillary. For disassembly, methods I and II were applied with dilution/disassembly flow rates of 150/250 and 250/150 μL min^–1^, respectively.

See Supporting Information Sections S-I for further information on the sample preparation methods (Figure 1).

### Chromatographic Conditions

#### Instrumentation

All experiments were performed on an Agilent 1290 Infinity 2D-LC system (Agilent, Waldbronn, Germany). The system consisted of one Infinity II high-speed pump (G7120A, Pump 1), one 1260 Infinity nano pump (G2226A, Pump 2), one Infinity isocratic pump (G1310A, Pump 3), and one Infinity binary pump (G4220A, Pump 4) with a Jet Weaver V100 mixer (100 μL internal volume) and with an 1100 series autosampler (G1329A). The system was equipped with two DADs: one Infinity II DAD (G7117B, DAD 1) and one Infinity DAD (G4212A, DAD 2) equipped with an Agilent Max-Light Cartridge cell (10 mm path length, *V*_det_ = 1.0 μL). The 6-port-2-position valve (P/N: 5067-4241, Valve 1) was installed in a valve drive (G1170A, S/N: DEBAD05428). Furthermore, the system was equipped with one Infinity thermostated column compartment (G1316C). This compartment contained a valve drive (G1170A) with an 8-port-2-position modulation valve (G4236A, Valve 2). The injector needle drew and ejected at a speed of 200 μL min^–1^.

For disassembling the particles, an Analytical Scientific Instruments mixer (P/N: 411-0150, 150 μL internal volume) was used. For mixing the disassembled effluent with a dilution flow, a Jet Weaver V100 mixer (100 μL internal volume) was used. All tubing and connections were made of stainless steel. The system was operated with Agilent OpenLAB CDS ChemstationEdition software (Rev. C.01.09).

The ^1^D column was an Agilent PL–PSDA cartridge type-2 (800 × 7.5 mm^2^ i.d., *d*_p_ 15 μm P/N: PL0850-1020). The ^2^D column was an Agilent ZORBAX RRHD Eclipse Plus C18 column (50 × 2.1 mm^2^ i.d., *d*_p_ 1.8 μm, 95 Å particles, P/N: 959757-902). Two Phenomenex SecurityGuard ULTRA guard-column holders (P/N: AJ0-9000) with each a UHPLC C18 cartridge (2.0 × 4.6 mm^2^ i.d., P/N: AJ0-8768) were used for trapping.

During method development, adjustments were made to the setup. The first 2D-LC results were obtained when Mixer 2 consisted of a Waters zirconia mixer (P/N: 700002911, 50 μL internal volume), and Valve 1 was an 8-port-2-position valve (P/N: 5067-4214). Instead of having it installed in a separate valve drive, the valve was placed in an Infinity thermostated column compartment (G1316C). For the 1D RPLC experiments, a high-pressure autosampler was used (G7129B). During SPAM optimization, smaller UHPLC C18 cartridges (2.0 × 2.1 i.d., P/N: AJ0-8782) were used.

See Supporting Information Sections S-II for further information on the configuration of the chromatographic system.

#### One-Dimensional HDC

In the one-dimensional HDC experiments, the mobile phase consisted of HDC eluent, and the flow rate was controlled via a flow program. The applied HDC conditions are based on earlier work by Pirok *et al.*(18,27) The program started at 1000 μL min^–1^ and decreased to 100 μL min^–1^ between 12.39 and 12.40 min and maintained this flow rate until the end of the run. At 12.40 min, the column effluent was sent to DAD 2 via a restriction capillary. The total analysis time was 35 min. The injection volume was 100 μL for the curcumin, Sudan-IV, and PLGA B NPs, and 50 μL for the PLGA NP A. Absorbance spectra were collected from 190 to 600 nm at frequencies of 20 and 10 Hz for DAD 1 (elution) and DAD 2 (waste), respectively.

#### One-Dimensional RPLC

For the one-dimensional RPLC experiments, mobile phase A was water with 0.1% FA (pH 2.6), and mobile phase B was ACN with 0.1% FA. The flow rate was set at 1000 μL min^–1^, and the gradient started at 70% B. From 0.10 to 0.40 min, mobile phase B increased from 70 to 100%. The composition was held at 100% B until 0.80 min. At 0.81 min, the composition decreased to 70% B. The total analysis time was 1.00 min. The injection volume was 1 μL for the calibration samples and 5 μL for the pre-disassembled NP samples. The DAD detector collected absorbance spectra from 190 to 600 nm at 10 Hz.

#### Nephelometry

For the nephelometry measurements, a Varian Cary Eclipse Fluorescence Spectrophotometer was used. The excitation wavelength was set at 650 nm, and the emission spectrum was recorded from 600 to 700 at 600 nm/min. The excitation and emission slit widths were set to 2.5 and 5.0 nm, respectively. The excitation and emission filters were set at auto and open, respectively.

#### Online Comprehensive HDC × RPLC and Online NP Disassembly

In the final HDC × RPLC setup, Pump 1 isocratically pumped HDC eluent according to the 1D-HDC method. From 12.09 min, Pump 2 and Pump 3 did not provide any flow. From 12.10 min until 35.00, Pump 2 and 3 were pumping ACN (0.1% FA) and water (0.1% FA, pH 2.6), respectively. Exact flow rates of these pumps varied per method and more details can be found in Table S6. For the final experiments, the 30ACN method was applied to all NPs, except for the Sudan-IV NPs for which the 60ACN method was applied. The ^2^D pump (Pump 4) started pumping at 12.10 min at a flow rate of 1000 μL min^–1^. The modulation time was 60 s. The RPLC method is described above (1D RPLC). The total run time of the 2D-LC method was 35.00 min. The injection volume for all samples was 100 μL, except for PLGA NP A solution of which 50 μL was injected.

#### Data Treatment

For the calculation of the peak areas for the nephelometry data, solvent optimization, and RPLC results, the **trapz** function from MATLAB R2022b was used. Horizontal baseline correction was performed on all HDC and RPLC chromatograms. Also, a Savitsky-Golay filter (3rd order, 21 framelength) was applied to all chromatograms using the **sgolayfilt** function. For the 2D-LC data, modulations were extracted by finding the apex of the valve switch peaks and reshaping the data by stacking these modulations. The **findpeaks** function from MATLAB 2022b was used to find these peaks tops. The resulting areas from the RPLC runs were further processed and visualized in histograms with Microsoft Excel. Supporting Information section S-IV shows more details on the RPLC calibration. MOREDISTRIBUTIONS (v 1.11) was used to plot the 2D-LC chromatograms and to perform the HDC size calibration.^35^ For this, a blank subtraction was performed, and the baselines of the chromatograms were horizontally aligned.

## Results and Discussion

### Design of the Comprehensive 2D-LC Method

#### One-Dimensional Separations

HDC was used for the separation of intact NPs. In HDC, the elution volume is often expressed as τ, where τ = 1 for *t*_0_.^16^ The actual analyte separation in HDC generally occurs between 0.8τ and 1.0τ. A comprehensive HDC × RPLC strategy was developed in which only the relevant part of the ^1^D effluent (*i.e*., comprising the size distribution of the intact NPs) was transferred to the ^2^D separation. For this, a flow program was used as described in more detail in S–III. The HDC dimension was calibrated on particle size using polystyrene nanosphere standards that were measured in triplicate. To ensure that the full HDC separation space was subjected to the second dimension, elution of both the largest and smallest calibrant (900 and 30 nm, respectively) was confirmed (Figure S1).

Figure S2 shows the HDC separation of the four NP samples. Each NP sample shows a unique size distribution. After calibration, the time axis was converted to size and the average size of the NP samples was determined (Tables S2 and S3).^35^ The average sizes were found to be 541, 129, 59, and 132 nm for PLGA NP A, PLGA NP B, curcumin-loaded PLGA–PEG-PLGA, and Sudan-IV-loaded PLGA–PEG-PLGA NP, respectively. For PLGA NPs A and B, a clear difference in particle size is observed, which is in agreement with the descriptions from the vendor. For NP B and the curcumin NP, an extra peak is observed besides the main peak, corresponding to a particle size of about 900 nm. This shows that HDC is capable of separating aggregates from the main NP distribution. Interestingly, despite being produced with the same NP precipitation method, the curcumin and Sudan-IV NPs show different particle size distributions. All HDC chromatograms of the NPs have been recorded at two wavelengths: 254 nm and the maximum absorbance wavelength of the dye (λ_max_) (Figure S3). As the polymer itself does not have a chromophore, the signal at 254 nm mostly originates from light scattering by the NP. Signals recorded at the λ_max_ of the encapsulated compound are a combination of the absorbance of the dye and the scattering of the NP. Notably, the scattering signal is most intense when the NP size is equal to the monitored λ (*i.e*., a 300 nm NP has the highest scattering signal at a λ of 300 nm). For NP samples A and B and the curcumin NP, the profiles at the two wavelengths are similar (note that the peak at *t*_0_ is only visible in the 254 nm trace). However, the profiles for the Sudan-IV NP are different for the two monitored wavelengths. The signal at λ_max_ clearly shows a trimodal distribution, while this is much less apparent at the 254 nm signal. Comprehensive sampling over the HDC distribution and subsequent analysis of the content by RPLC would reveal the particle contents as a function of this distribution.

A 1 min RPLC method was developed for the separation of the three dyes (S–IV) to allow for short modulation times in the second dimension. Figures S4 and S5 show the RPLC separation and online-recorded UV spectra of the three dyes, respectively. Since the encapsulated compounds have a relatively high hydrophobicity, the RPLC gradient started at a mobile phase composition of 70% ACN. Calibration lines were established by measuring a mixture of the three dyes at 8 different concentrations, each using 5 technical replicates. As described in Supporting Information Section S-II, the RPLC experiments were performed with the SPAM setup. The RPLC-UV/vis analysis was calibrated with dye samples of concentrations ranging from 0.5 up to 20 mg L^–1^ (Figure S6). The average LOD/LOQ values were 0.28/0.83, 0.42/1.27, and 0.47/2.80 mg L^–1^ for curcumin, coumarin-6, and Sudan-IV, respectively (Table S4).

#### Disassembly of the Nanoparticles

**Figure 2:**
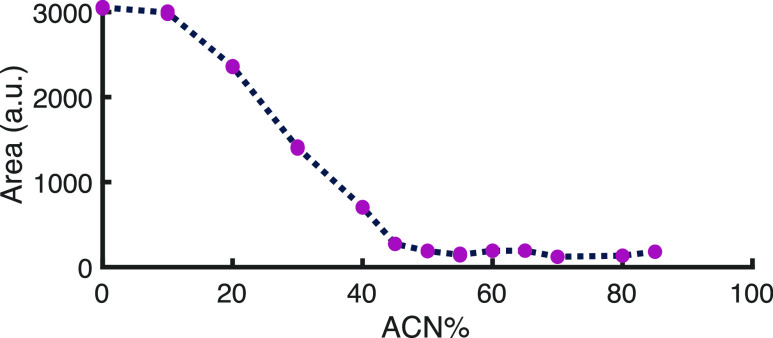
Nephelometry results of PLGA–PEG-PLGA NPs suspended in mixtures of HDC eluent and ACN. The *x*-axis depicts the percentage of ACN in the solvent, and the *y*-axis the observed scatter signal intensity. The means of the 5 technical replicates are plotted as purple points.

The intact PLGA NPs with the encapsulated cargo are commonly suspended in an aqueous medium. To assess the encapsulated content, the NPs must be disassembled to have both the polymer and cargo in solution for further analysis. An organic solvent was used to disintegrate the NPs and to prevent the released dyes from precipitating, as their solubilities in pure water are very low. ACN was pragmatically chosen as the disassembly solvent because it dissolves the dyes and can also serve as an organic modifier in the mobile phase for the ^2^D RPLC separation. To determine the minimum percentage of ACN in water that is needed for NP disassembly, two approaches were subsequently followed (Supporting Information S-V). Both methods rely on the detection of light scatter induced by intact NPs. In the first simple approach, the particle disassembly was visually assessed by observing the accompanying decrease in sample cloudiness and reduction of scattered light intensity when a red laser beam is shined through the sample vial (Figure S7). These results suggested that disassembly occurs at about 50% ACN. For a more accurate determination of the ACN percentage that is needed for NP disassembly, nephelometry was used. This technique measures the intensity of light scattering by a solution or suspension using a fluorescence spectrophotometer.^36,37^ For NPs in suspension, Mie scattering is expected to be dominant.^38^ This type of scattering is caused by relatively large particles and will therefore contribute only to the nephelometry signal when samples contain intact NPs. Rayleigh scattering predominantly comes from the solvent molecules and is therefore expected to be virtually constant for each NP sample. Consequently, a decrease in the scattering intensity is expected when the concentration of intact NPs diminishes. PLGA–PEG-PLGA NP suspensions were exposed to mixtures of ACN and HDC eluent at different volumetric ratios, while the NP concentration was kept constant to allow comparison of the measured scatter intensities (Table S5). Figure 2 shows the results from the respective nephelometry experiments (blank measurements are included in Figure S8).

In Figure 2, a high plateau is observed at low ACN%, where the NPs are still intact. The scatter signal intensity starts to decrease when the ACN concentration exceeds 10%, indicating that the NPs are disintegrating. The signal gradually decreases until a minimum plateau is reached, starting at about 50% ACN. Here, the scatter intensity is similar to that observed for blank solutions with no NPs. These results are in good agreement with the results from the visual experiments (Figure S7). The same trend was observed when the experiments were performed in ACN/water mixtures (Figure S8). Overall, the obtained results indicate that at least 50% ACN is required to fully disassemble the NPs.

### Development of HDC × RPLC Setup Using Dye-Loaded PLGA NPs

Based on the developed 1D methods and disassembly assessment of the NPs, the HDC × RPLC system was designed, as depicted in Figure 1. The aqueous effluent of the HDC column was mixed online with ACN using Pump 2, and subsequently diluted with water using Pump 3 to allow trapping of the analytes (see below). As described before, only the relevant ^1^D elution window (*i.e*., containing the HDC distribution of the NPs) was comprehensively fractioned to the ^2^D. During fractionation, the flow rate in the ^1^D and ^2^D were constant and their rates were maximized to achieve an overall fast 2D-LC method. In comprehensive 2D-LC, the total analysis time is heavily dependent on the ^2^D time, which is restricted by column pressure limitations. Fast RPLC was used in ^2^D to achieve modulation times of 60 s at 1000 μL min^–1^. The maximum pressure of the ^1^D column (120 bar) was limiting the maximum flow rates that could be provided by Pumps 1, 2, and 3. Consequently, the ^1^D flow rate could not exceed 1000 μL min^–1^ for the fast-flow step and 400 μL min^–1^ for the combined disassembly/dilution flow rate during NP elution. To ensure complete NP disintegration, a disassembly flow rate resulting in 60% ACN was chosen. This was achieved when an ACN disassembly flow rate of 150 μL min^–1^ (Pump 2) was mixed with the 100 μL min^–1 1^D flow rate (Pump 1). This method is referred to as Method A.

**Figure 3:**
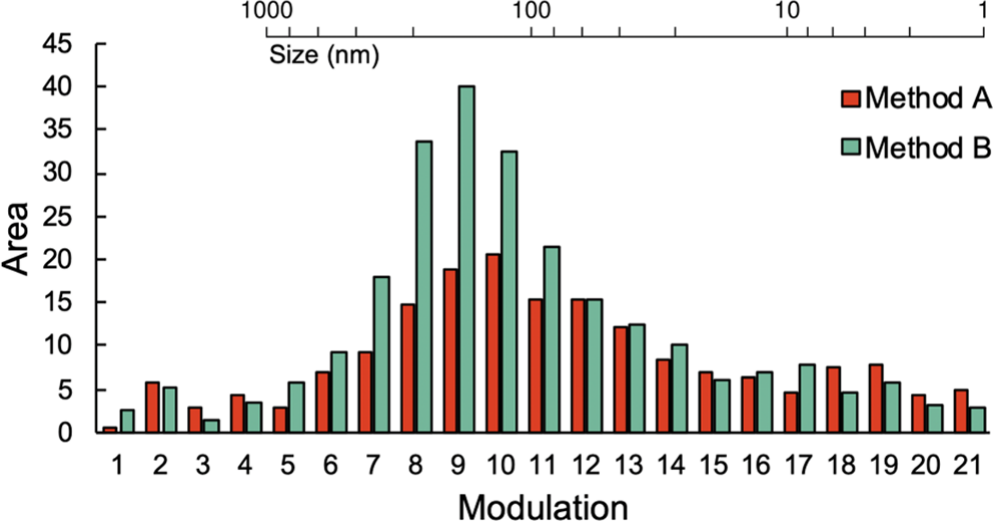
Peak areas per modulation as obtained during HDC × RPLC of Sudan-IV. Dilution flow method (A) 250 μL min^–1^ and (B) 100 μL min^–1^. A scale showing the corresponding NP size is included in the plot.

For comprehensive fractionation of the HDC effluent, stationary-phase-assisted modulation (SPAM) was applied. In SPAM, small columns are used to trap the analytes and change from the ^1^D to the ^2^D eluent composition.^39,40^ After NP disassembly, a dilution flow of aqueous solvent (Pump 3) had to be introduced to achieve the trapping of the analyte on the RP trap columns. For Method A, the dilution flow was set at 250 μL min^–1^. The total HDC × RPLC analysis composed a total of 21 complete ^2^D modulations. Modulations 1 and 21 started at 13.5 and 33.5 min, respectively, making the total analysis time 34.5 min. Figure 3 (Method A) shows the results obtained for the HDC × RPLC analysis of Sudan-IV. The areas of the Sudan-IV peak observed in ^2^D were integrated and plotted for each successive modulation. Because these modulations correspond to the ^1^D HDC separation, the amount of encapsulated compound over the HDC distribution is obtained. The corresponding 2D-LC plots are shown in Supporting Information S-VI Figure S9.

When the dilution flow was decreased to 100 μL min^–1^, the Sudan-IV peak areas corresponding to the middle of the HDC distribution increased (Figure 3, Method B). We suspected that precipitation of the dye occurred during the application of a dilution flow of 250 μL min^–1^. As Sudan-IV is the most hydrophobic of the three test dyes, it is most prone to precipitation when the percentage ACN is too low after the addition of the aqueous dilution flow. With Method A, the resulting ACN concentration was 30%, whereas with Method B, it was around 40%. During the first exploring HDC × RPLC experiments, we also observed carryover of the more hydrophobic dyes (coumarin-6 and Sudan-IV) when the percentage ACN was relatively low, suggesting that the dyes precipitate in the system. Clearly, for proper performance, the dilution flow of water should not be too high. However, the overall percentage of organic solvent entering the trap columns may affect the trapping efficiency of the dyes, which also should be considered. Therefore, further attention was paid to the mutual optimization of these parameters, as will be discussed in the section below.

### Optimization of Online Disassembly and Modulation

**Figure 4:**
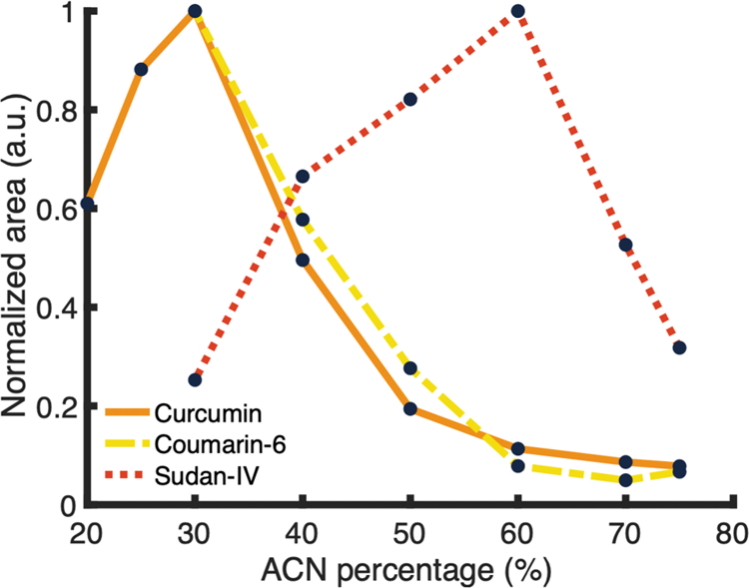
Normalized peak areas for the dyes obtained with RPLC after online disassembly of the respective NPs as a function of the percentage of ACN that is introduced to the trap columns (*i.e*., resulting from mixing the ^1^D, disassembly, and dilution flow).

To optimize the solvent modulation of the 2D-LC method while also taking into account the online NP disassembly that needs to take place, the following parameters were considered: (i) the ACN disassembly percentage, (ii) the total flow rate (hence, volume) entering the trap columns, and (iii) the ACN percentage of the solvent introduced to the trap columns. Based on the previous experiments, it was concluded that an ACN percentage of at least 50% (v/v) in the disassembly solvent ensured complete disintegration of the NPs. For the optimization study, the 2D-LC setup was used but without the HDC column in place. A DAD was placed after the RPLC column (“elution detector”) and another DAD was installed to monitor the effluent of the trap columns during loading (“waste detector”; see Figure 1). The ^1^D and ^2^D flow rates were kept constant at 100 (Pump 1) and 1000 (Pump 4) μL min^–1^, respectively. The sum of the disassembly (ACN) and dilution (water) flow rates was kept constant at 400 μL min^–1^, but the ratio between these two flows was alternated. The overall flow rate (*i.e*., ^1^D + disassembly + dilution flow) directed to the trap columns was always 500 μL min^–1^. This way, the disassembly ACN percentage varied between 50 and 79%, while the ACN percentage entering the trap columns varied between 20 and 75%. A total of 8 ratios were tested; more details are provided in Table S6. Solutions of each NP were injected into the system, and the dye peaks subsequently obtained with RPLC were integrated and their areas normalized to the largest peak and plotted against the percentage ACN entering the trap columns (Figure 4).

Figure 4 shows the signal from the elution detector at different ACN% entering the traps (see Supporting Information S-VII for more details). The signal increases with increasing ACN%, until an optimum is reached (30 and 60% for curcumin/coumarin-6 and Sudan-IV, respectively). After this optimum, the signal intensity decreases with increasing ACN%. Figure S10 shows separate traces from the elution and waste detectors for each method. When comparing the elution and waste detector signals, an inversely proportional trend is observed. With increasing ACN% after the optimum, a signal increase in the waste detector is observed, while the signal in the elution detector is decreasing. This indicates that the analyte is not sufficiently trapped meaning that breakthrough from the trap columns occurred. This corresponds with the results from Figure 3 in which an increased dilution F (*i.e*., a lower ACN% on the traps) improved the signal intensity.

However, at lower ACN%, the signal in the elution detector also increases while no signal is observed in the waste detector. This cannot be attributed to breakthrough but to precipitation of the analyte. The total ACN% entering the traps should be sufficiently high to dissolve the hydrophobic dyes. At too low ACN%, the dyes do not fully dissolve and precipitate in the system, resulting in no signal in both detectors. This is particularly unwanted, as this precipitation could be destructive to the system. These experiments demonstrate the importance of optimizing the ratio between disassembly and dilution flow. Retention modeling could be applied in future work to predict optimal trapping conditions and facilitate method development.^41^

### Optimized HDC × RPLC Separation of Dye-Loaded Polymeric NPs

**Figure 5:**
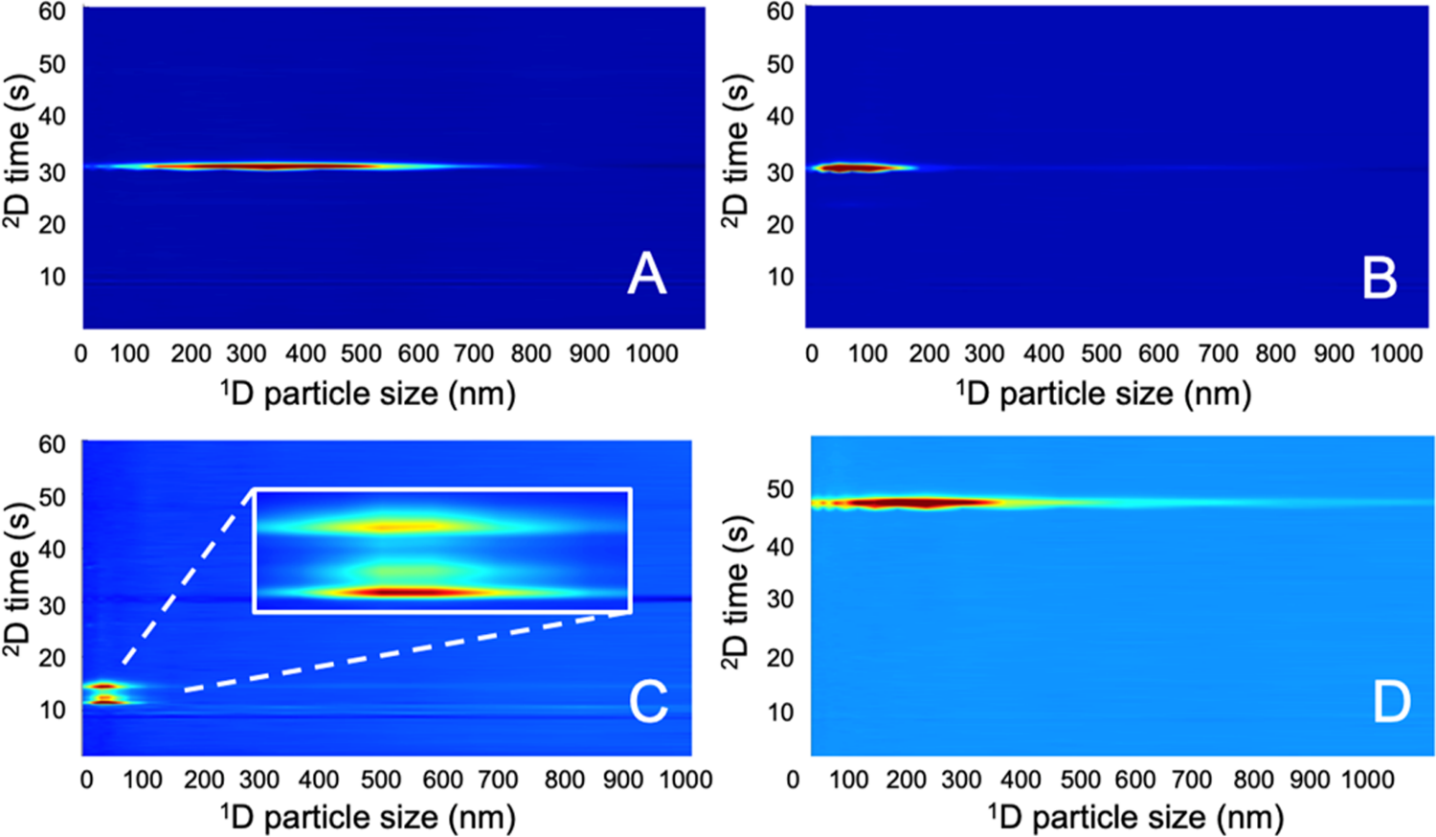
Contour plots obtained during HDC × RPLC of dye-loaded NPs. (A) PLGA NP A, (B) PLGA NP B, (C) curcumin-loaded NP (zoom showing 0 to 80 nm range), (D) Sudan-IV-loaded NP. The *x*-axis depicts the ^1^D separation as a function of particle size and the *y*-axis shows the ^2^D RPLC separation as a function of retention time.

The HDC × RPLC system was applied under the optimized conditions for the analysis of the four different NP samples (Figure 4). PLGA NPs A, B, and the curcumin NPs were analyzed with the 30% ACN method and the Sudan-IV NPs were measured with the 60% ACN method. Figure 5 shows the 2D-LC chromatograms that were obtained for the four samples.

The 2D-LC plots show the encapsulated dye over the particle size distribution. The obtained distributions are in agreement with the 1D HDC experiments. The optimized solvent conditions were applied, and indeed, no breakthrough of the analyte was observed in the waste detectors (Supporting Information S-VIII, Figures S11–S18). The 2D-LC results of NP B do not show a signal at the retention time of the NP aggregate that has been detected in 1D HDC. This suggests that the aggregate does not contain coumarin-6.

While most analyzed NPs show a consistent single peak in the ^2^D separation, the curcumin NP shows three peaks (Figure S16). The first- and second-eluting peaks show very similar UV spectra, whereas the UV spectrum for the third-eluting peak is different (Figure S16). As curcumin is a temperature- and light-sensitive compound, we hypothesize that the extra peaks are caused by the degradation of curcumin. In preliminary experiments (not shown), we observed changes in the peak ratios when the curcumin NPs were measured over time. More research is needed for monitoring the degradation of curcumin and possibly other NP loads, but this result indicates that the method could provide information on the stability of payloads in formulations.

**Figure 6:**
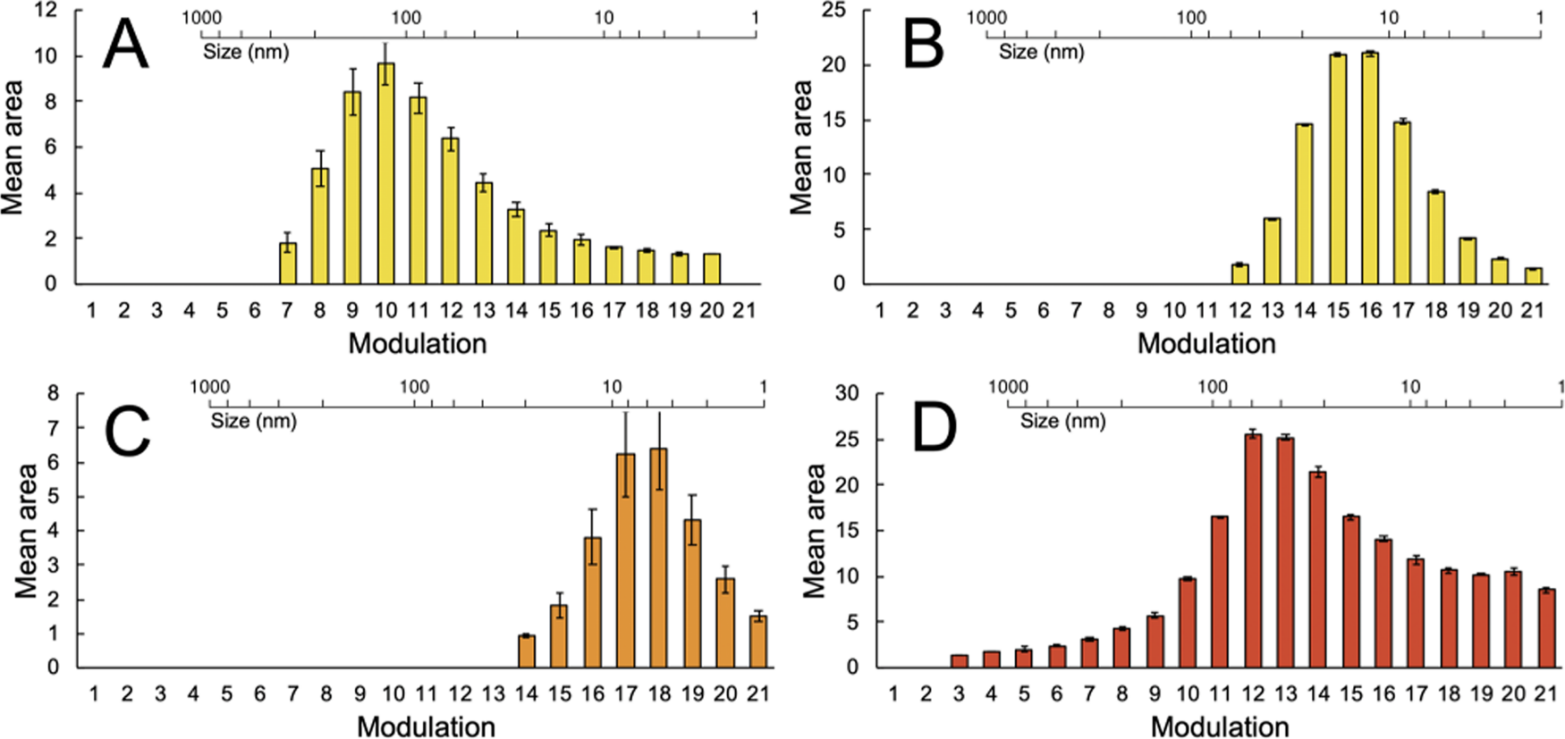
Mean dye-peak areas per modulation as obtained during HDC × RPLC of dye-loaded NPs. (A) PLGA NP A, (B) PLGA NP B, (C) curcumin-loaded NP, and (D) Sudan-IV-loaded NP. Each sample was measured in triplicate. Error bars indicate the corresponding standard deviations. Scales showing the corresponding NP size are included in the plots.

The feasibility of using the developed HDC × RPLC method for quantitation of the encapsulated cargo was also investigated. For the quantification of the ^2^D modulations, the LOQ values per dye were used as a threshold value. The mean areas of the dye peak per modulation and the corresponding standard deviations are plotted in Figure 6. Note that the modulation number is a time- and not a size-dimension. Therefore, these results follow the HDC elution order (*i.e*., earlier modulations correspond to larger particle sizes).

The elution profiles for the dye samples correspond to the results in Figure 5. In contrast to the 1D HDC results reported in Figure S3, in which the signals are convoluted (*i*.*e*. resulting from both dye absorbance and NP scattering), the signals in Figure 5 correspond to the actual amount of cargo as a function of particle size. As observed before, no coumarin-6 was detected for the NP aggregate of PLGA NP B. Using the earlier established calibration curves, the concentration of released dye was calculated by taking the sum of the mean areas per modulation and correcting for the *V*_inj_ of the sample (Table S7). The coumarin-6 concentrations in NPs A and B were 1.15 ± 10% and 0.96 ± 1% mg L^–1^, respectively. For the curcumin and Sudan-IV NPs, the dye concentrations were 0.27 ± 20% and 1.99 ± 2% mg L^–1^, respectively. As expected, commercial PLGA NPs show similar dye loads. Although the same in-house protocol was used to formulate the curcumin and Sudan-IV NPs, the curcumin concentrations of the free dye were significantly lower. Furthermore, the standard deviation of 20% for the determined concentration of curcumin was relatively high. As the breakthrough of curcumin was not observed, another explanation for the relatively low signal of curcumin was suspected.

Possibly, not all curcumin had been encapsulated in the NPs, meaning that nonencapsulated dye would be present (*i.e*., free dye). When very hydrophobic, such as for Sudan-IV, this free dye would precipitate, as it does not dissolve in the aqueous sample solvent. However, curcumin is less hydrophobic and is fairly soluble in water. Dissolved free dye is expected to elute at *t*_0_ in HDC, along with other small molecules. As we did not observe a signal for curcumin at *t*_0_, we hypothesized that curcumin might have been retained on the HDC stationary phase. In principle, interaction with the stationary phase should be prevented by using a mobile phase comprising salts and surfactants.^16^ While the NPs did not retain and showed normal HDC behavior, the dyes, which are small molecules, could still adsorb to the polar stationary phase of the HDC column. In order to check for chromatographic retention of curcumin in HDC, we ran an extended HDC × RPLC method that also comprehensively fractioned after the HDC *t*_0_ (Supporting Information S-IX). This resulted in a long 2D-LC method comprising 225 modulations. Indeed a large curcumin band was observed after 125 min starting at the 113th modulation (Figure S19). The dye signals from the modulations were quantified and summed, resulting in a total injected concentration of 5.79 mg L^–1^ of curcumin. Although an HDC artifact, we suggest that the retention in HDC could be exploited for the differentiation between encapsulated and free API (*i.e*., determination of the encapsulation efficiency), yet this should be investigated more thoroughly in a future study.

## Conclusions

We have developed a comprehensive 2D-LC method for the simultaneous analysis of the size and encapsulated cargo of dye-loaded NPs. HDC and RPLC were used for ^1^D and ^2^D separations, respectively. Nephelometry was used to assess NP disassembly conditions for the sample transformation step. Solvent conditions were optimized to ensure complete disassembly of NPs and to minimize the risk of precipitation and ^2^D analyte breakthrough. Furthermore, the released dyes in the ^2^D RPLC modulations were quantified per modulation. The final HDC × RPLC system shows great potential for the multidimensional characterization of medicinal NPs. The system is MS-compatible and allows for a multidetector approach. This would be especially useful for the assignment of impurities and degradation products. Nevertheless, our method demonstrates comprehensive characterization of loaded NPs and would be a useful tool for stability studies and multiattribute DDS analysis.

## Supporting Information Available

1D separation dimensions, transformation interface, and optimization of the resulting LC × LC separations (PDF)
