# Supplementary material for: Characterization of Dye-Loaded Poly(lactic-co-glycolic acid) Nanoparticles by Comprehensive Two-Dimensional Liquid Chromatography Combining Hydrodynamic and Reversed-Phase Liquid Chromatography
Source: Anal Chem. 2023 Dec 13;95(51):18767–75. doi: 10.1021/acs.analchem.3c03356 (PMC10753526; doi:10.1021/acs.analchem.3c03356)
Supplement: Supplementary file 1 — ac3c03356_si_001.pdf [file ac3c03356_si_001.pdf]

SUPPORTING INFORMATION

Characterization of dye-loaded poly(lactic-*co*-glycolic acid) nanoparticles by comprehensive two-dimensional liquid chromatography combining hydrodynamic and reversed-phase liquid chromatography

Joshka Verduin[1,2*], Luca Tutiš[1,2], Alexander J. Becking[1,2], Amin Famili[3], Kelly Zhang[3], Bob W.J. Pirok[2,4], Govert W. Somsen[1,2]

[1] Vrije Universiteit Amsterdam, Department of Chemistry and Pharmaceutical Sciences, Amsterdam Institute of Molecular and Life Sciences, Division of BioAnalytical Chemistry (AIMMS), De Boelelaan 1085, 1081 HV Amsterdam, the Netherlands
[2] Centre of Analytical Sciences Amsterdam (CASA), Science Park 904, 1098 XH Amsterdam, the Netherlands
[3] Synthetic Molecule Pharmaceutical Sciences, Genentech, Inc., 1 DNA Way, South San Francisco, CA 94080, United States
[4] University of Amsterdam, van 't Hoff Institute for Molecular Sciences (HIMS), Analytical-Chemistry Group, Science Park 904, 1098 XH Amsterdam, the Netherlands

TABLE OF CONTENTS

S-I Preparations **2**
S-II Configuration of the chromatographic system **2**
S-III Hydrodynamic chromatography **3**
S-IV Reversed-phase liquid chromatography **6**
S-V Offline NP disassembly **7**
S-VI Development of HDC × RPLC set-up using Sudan-IV-loaded PLGA-PEG-PLGA NPs **8**
S-VII Optimization of online NP disassembly and modulation **9**
S-VIII Optimized HDC × RPLC analysis **10**
S-IX Extended HDC × RPLC separation of curcumin-loaded PLGA-PEG-PLGA NP **14**

S-I Preparations

For the HDC size calibration, the polystyrene nanospheres were diluted 100-fold in HDC eluent. The fluorescent coumarin-6 loaded PLGA NPs A and B were first suspended 5 times in water, after which it was further diluted 10-fold in water to a final concentration of 0.1% (w/v).

PLGA-PEG-PLGA NPs were synthesized via the nanoprecipitation method.[27] Stock solutions of PLGA-PEG-PLGA (6000 mg L^{-1}) and dye (curcumin or Sudan-IV, 500 mg L^{-1}) were prepared in acetone. PLGA-PEG-PLGA stock (200 μL) was mixed with dye stock (100 μL). The polymer/dye mixture was then suspended in water (4 mL), after which it was sonicated for 15 min. Next, the sonicated suspension of the dye-loaded NPs was placed in a vacuum oven at 38°C for 5 hr. This resulted in a colored (yellow for curcumin and red for Sudan-IV) and turbid suspension.

The samples for offline disassembly and nephelometry were prepared by mixing 125 μL of PLGA-PEG-PLGA NP in acetone (1200 mg L^{-1}) with 875 μL solvent. This solvent consisted of a mixture of ACN and HDC eluent at ratios varying from 0/100 to 85/15 (ACN/HDC eluent, v/v) with a 5% or 10% increment of ACN. The same concentration of polymer was present in each sample. Exact compositions per sample are reported in Table S5.

For RPLC optimization, mixtures of curcumin, coumarin-6, and Sudan-IV were prepared in ACN in the concentrations 0.5, 1.0, 2.0, 2.5, 5.0, 10.0, 15.0, and 20.0 mg L^{-1} each. Pre-disassembled NP samples were prepared by diluting the dye-loaded NP samples 10-fold in ACN (v/v).

S-II Configuration of the chromatographic system

The full HDC×RPLC set-up is schematically represented in Figure 1. Four different set-ups have been used; including a 1D HDC, 1D RPLC, online disassembly, and HDC×RPLC set-up. For 1D HDC measurements (Part A), Pump 1 was connected to the autosampler, after which the eluent was sent to the HDC column. Subsequently, the effluent was either sent to DAD 1 ('elution') or DAD 2 ('waste') with Valve 1.

For 1D RPLC experiments, Pump 4 was connected to the high-pressure autosampler, after which the effluent was sent to the SPAM set-up (Part C). The autosampler was connected to Valve 2, which was connected with with two trap columns, DAD 2, and the RPLC column. DAD 2 was used as a waste detector. The SPAM effluent was sent to the thermostatted column compartment with a 3-μL heating element and temperature control at 40°C. This effluent was analyzed with DAD 1. For each trap position a separate calibration line was obtained.

For the 2D-LC runs, Pump 1 was connected to the injector that was coupled to the HDC column. The effluent was sent to Valve 1, which sent the effluent either to waste or Part B. In Part B, the effluent of Valve 1 was connected to a stainless-steel T-piece. For this connection, a restriction capillary (0.95 m, 100 μm i.d.) was installed to avoid backflow of ACN into part A of the system. Pump 2 contained ACN and was also connected to the T-piece. The combined water/ACN flow was sent to Mixer 1. This was subsequently sent to Part C of the system. The Mixer 1 effluent was sent to Mixer 2, which was connected to Pump 3 containing aqueous eluent (water + 0.1% FA (pH 2.6)). The combined flow was then sent to Valve 2 on which the SPAM set-up was installed. The RPLC column was connected to the thermostatted column compartment with a set temperature of 40°C, as described above. Finally, DAD 1 was connected to the RPLC effluent and DAD 2 was installed as a SPAM waste detector.

During the online disassembly experiments, the full HDC×RPLC set-up has been used (parts A-C), however, no HDC column was installed, hence the injector was directly connected to Valve 1.

S-III Hydrodynamic chromatography

To decrease the analysis time of the first dimension, a flow program was applied to the HDC separations, in which a higher flow rate was used in the first part of the analysis. To facilitate this, a valve was installed after the HDC column that directed the HDC effluent to a waste detector during τ = 0.0-0.8 (i.e., from 0.00 until 12.39 min) at a flow rate of 1000 μL min^{-1}. At 12.40 min, the valve switched and the effluent was directed to a second detector at 100 μL min^{-1}. Polystyrene (PS) standards (Table S1) were used to calibrate the HDC dimension on particle size. The chromatogram below (Figure S1) shows elution of all polystyrene standards within the flow program, demonstrating that no NPs will be lost during comprehensive HDC×RPLC. For preprocessing, the baselines of the chromatograms were first horizontally aligned at 14 min, followed by intensity normalization. Elution times from the peaks were calculated with the findpeaks function in MATLAB (MinPeakHeight =0.99 and NPeaks =1).

Table S1. Specification of the NIST polystyrene nanosphere standards used for calibration.

Standard	Mean diameter (nm)	SD (nm)	CV (%)
50	46	7.3	15.8
70	70	7.3	10.4
100	100	7.7	7.7
200	203	5.9	2.9
350	345	6.5	1.9
500	510	9.2	1.8
900	903	4.1	0.5

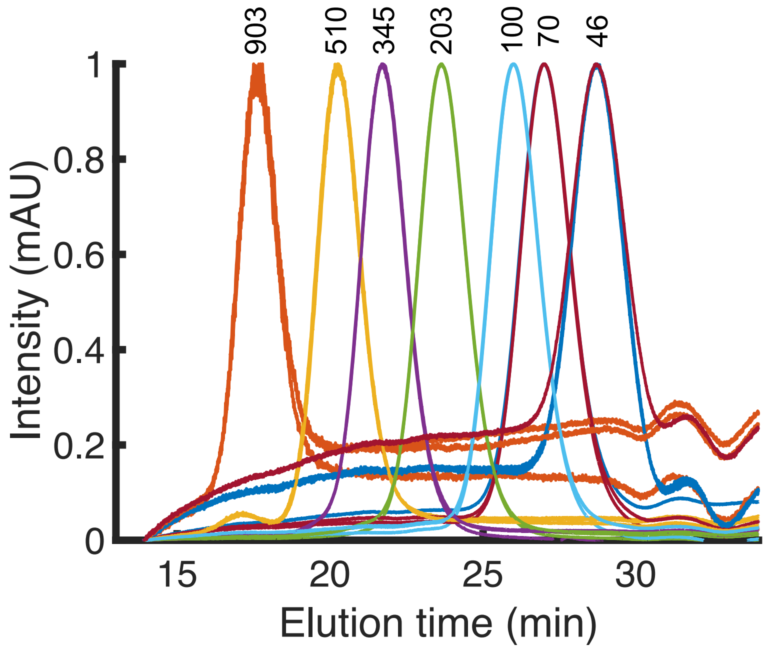

Figure S1. HDC chromatograms of polystyrene nanoparticle standards obtained using the flow program. The average sizes (nm) of the NP standards are reported at the tops of the corresponding peaks. Each sample was measured in 3 technical replicates.

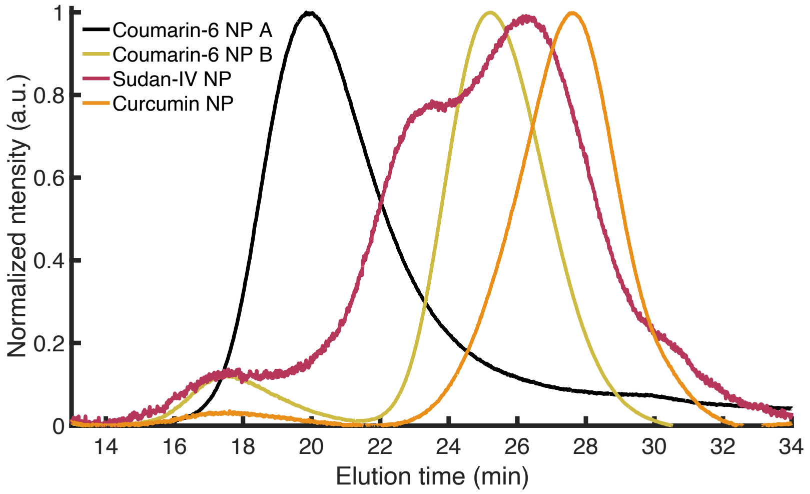

Figure S2. HDC chromatograms of dye-loaded polymeric nanoparticles with the same pre-processing (horizontal baseline alignment and normalization) as applied for the polystyrene NPs (Figure S1). Detection wavelength, 254 nm.

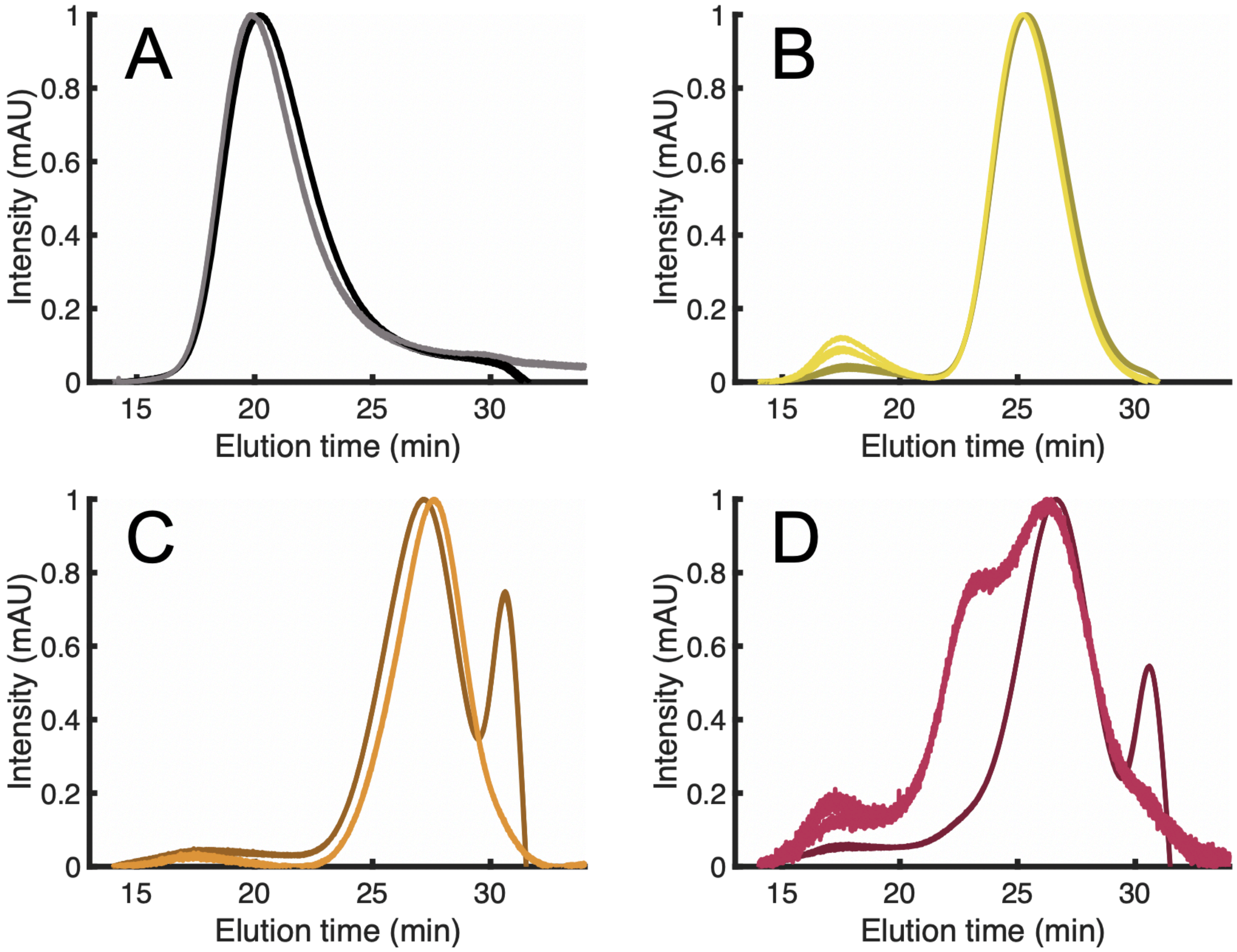

Figure S3. HDC chromatograms of dye-loaded polymeric NPs: (A) Coumarin-6 NP A, (B) Coumarin-6 NP B, (C) curcumin NP, and (D) Sudan-IV NP. The darker traces represent the chromatograms detected at 254 nm and the lighter traces represent the chromatograms recorded at the maximum absorption wavelength of the dye. Each chromatogram was recorded in 3-fold. Note that signal dip at 30 min in chromatograms C and D corresponds with t_0.

Table S2. Average elution times (t_E) for the polystyrene NP standards. These values and the NIST SD values (Table S1) were used in MOREDISTRIBUTIONS for the conversion from elution time to size.

Particle size (nm)	t_E average (min)
903	17.55
510	20.19
345	21.62
203	23.55
100	25.90
70	26.89
31	28.61

Table S3. Average sizes of the dye-loaded polymeric NPs resulting from MOREDISTRIBUTIONS. A threshold of 5% was applied in the software program. All NP samples were measured in 3-fold.

Sample	Average size (nm)
PLGA NP A	541
PLGA NP B	129
	888
Curcumin NP	59
Sudan-IV NP	132
	930

S-IV Reversed-phase liquid chromatography

The RPLC chromatograms were recorded at the maximum absorption wavelength of the encapsulated dye. All RPLC chromatograms were processed with MATLAB. All data was smoothed with a Savitsky-Golay filter with a 3rd order polynomial and a window width of 21. First vertical and then horizontal baseline corrections were applied. The trapz function was used to calculate the peak areas. For calibration, all samples were measured in 5-fold on both trap positions and separate calibration lines were calculated.

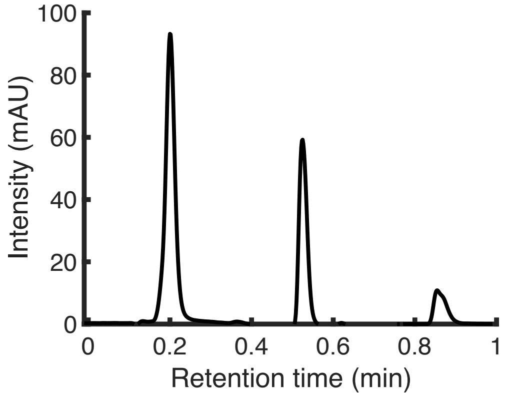

Figure S4. RPLC chromatogram of a dye mix (20 mg L^{-1}) recorded at 420 nm. The elution order is curcumin, coumarin-6, Sudan-IV.

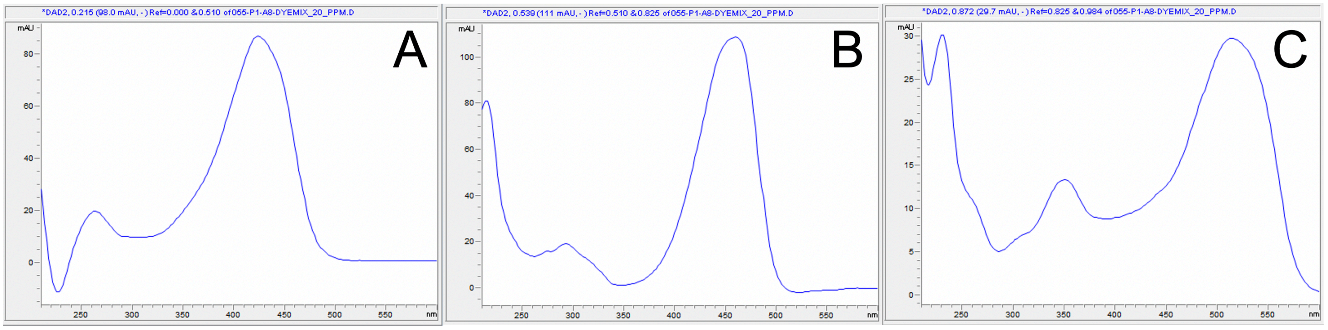

Figure S5. UV absorbance spectra of the three dyes obtained during LC-DAD (Figure S4). (A) curcumin, (B) coumarin-6, (C) Sudan-IV.

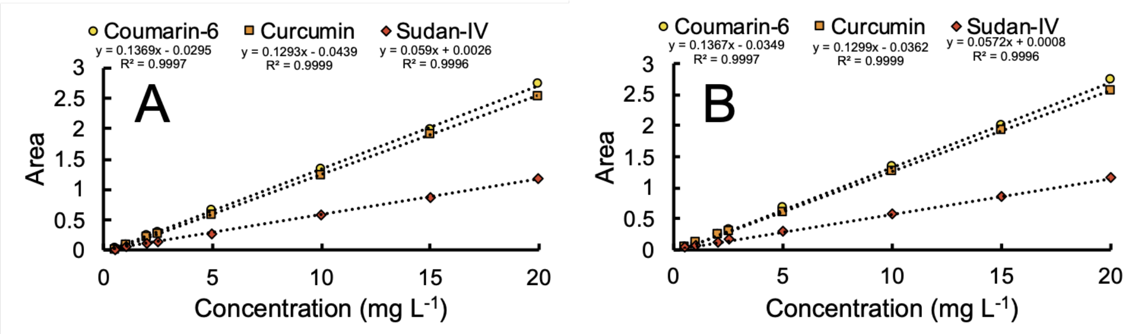

Figure S6. Calibration lines for the different dyes at trap positions 1 (A) and 2 (B) measured with trap-RPLC. Each concentration was measured in 5 technical replicates. The averages are shown in these calibration curves.

Table S4. Results of the trap-RPLC calibration. The reported values are the average areas from 5-fold repeats, from which 5 measurements were considered outliers and not included in the set. The limit of detection (LOD) and limit of quantification (LOQ=3*LOD) are reported for the different dyes per trap.

Analyte	LOD (mg L^{-1})		LOQ (mg L^{-1})	
	Position 1	Position 2	Position 1	Position 2
Curcumin	0.28	0.27	0.84	0.81
Coumarin-6	0.42	0.42	1.27	1.26
Sudan-IV	0.44	0.49	1.32	1.48

S-V Offline NP disassembly

Table S5. Preparation PLGA-PEG-PLGA NPs in different ACN-aq mixtures. The total concentration of polymer was 150 mg L^{-1} in all samples. Aq is either water or HDC eluent. ACN% and aq% show the total percentage of ACN and aqeous solvent inside the sample, respectively. NP (μL), ACN (μL), and aq (μL) show the volumes of NP suspension, ACN, and aq that have been used to prepare the disassembly samples.

ACN (%)	0	10	20	30	40	45	50	55	60	65	70	80	85
Aq (%)	100	90	80	70	60	55	50	45	40	35	30	20	15
NP (μL)	125	125	125	125	125	125	125	125	125	125	125	125	125
ACN (μL)	0	100	200	300	400	450	500	550	600	650	700	800	850
Aq (μL)	875	775	675	575	475	425	375	325	275	225	175	75	25

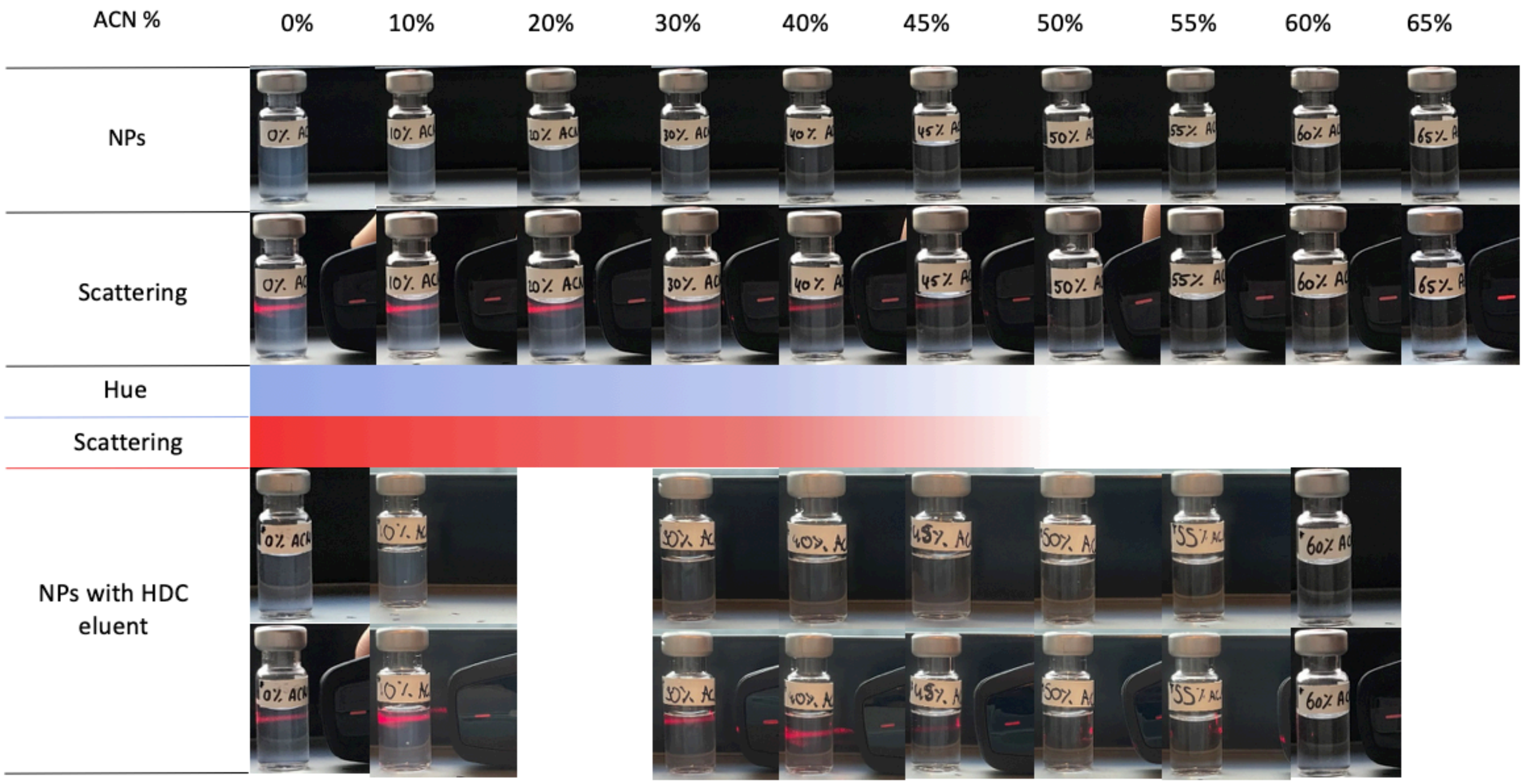

Figure S7. Visual observation of disassembly of PLGA-PEG-PLGA NPs. The top two rows and bottom two rows of pictures correspond to NPs that have been dissolved in water and HDC eluent, respectively. The 'Hue' and 'Scattering' represent quasi-quantitatively the visual sample turbidity and laser pointer scattering, respectively. This experiment suggests that full NP disassembly is achieved at 50% ACN.

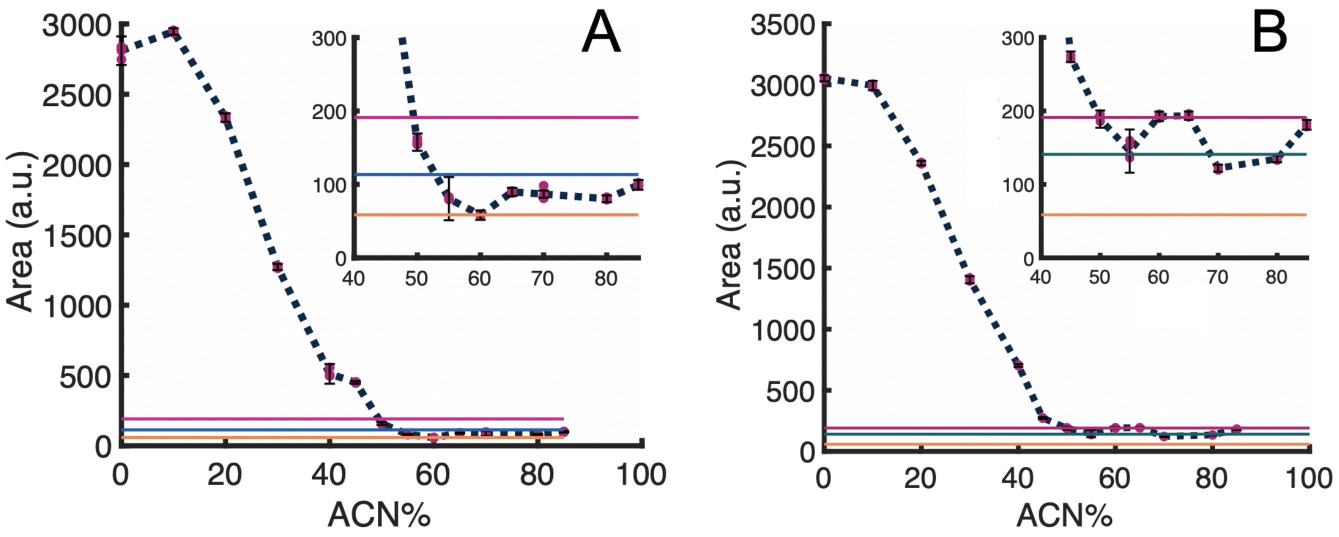

Figure S8. Nephelometry results for the off-line disassembly of PLGA-PEG-PLGA NPs using ACN. The nephelometry has been performed for both the water- (A) and HDC-diluted (B) samples. Each sample was measured in 5-fold. The horizontal lines show the signal for reference samples. The 95% confidence intervals are plotted. The pink line corresponds to PLGA-PEG-PLGA dissolved in ACN (150 mg L^{-1}), the blue one to aqueous solvent (water for A and HDC eluent for B), and the orange line to ACN.

S-VI Development of HDC × RPLC set-up using Sudan-IV-loaded PLGA-PEG-PLGA NPs

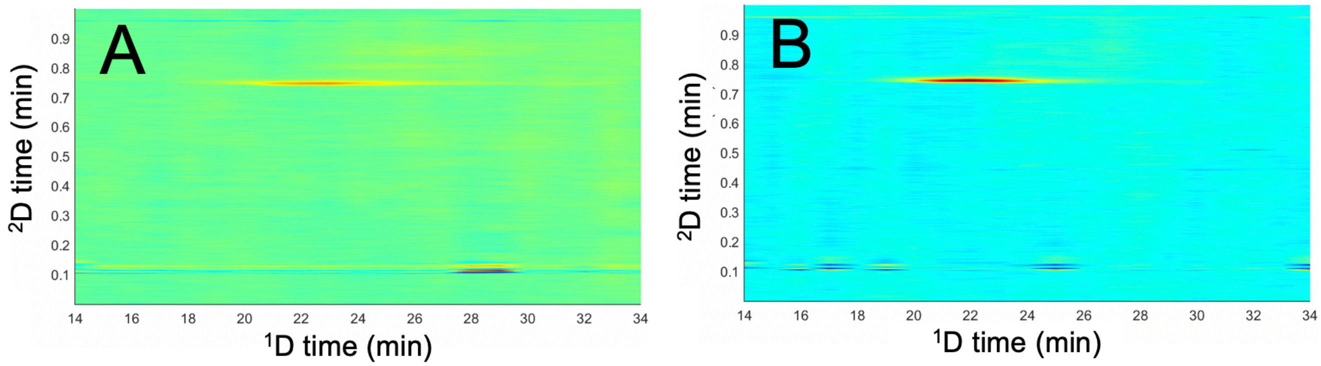

Figure S9. HDC × RPLC chromatograms of Sudan-IV-loaded PLGA-PEG-PLGA NPs. Dilution flow, (A) 250 and (B) 100 µL min^{-1}.

S-VII Optimization of online NP disassembly and modulation

Table S6. Chromatographic conditions for the online NP disassembly and modulation experiments. The ^{1}D and ^{2}D flow rates remained constant at 100 and 1000 µL min^{-1}, respectively, during modulation. The flow rates provided by the disassembly (ACN) and dilution (water) pumps were varied, however, the total flow rate of these pumps remained constant at 400 µL min^{-1}. The disassembly percentage ACN was at least 50% as earlier off-line experiments showed lower ACN percentages provided incomplete NP disintegration.

Method name	^{1}D HDC flow (µL min^{-1})	Disassembly flow (µL min^{-1})	Disassembly percentage ACN (%)	Dilution flow (µL min^{-1})	Overall percentage ACN to trap (%)	^{2}D RPLC flow (µL min^{-1})
20ACN	100	100	50	300	20	1000
25ACN	100	125	56	275	25	1000
30ACN	100	150	60	250	30	1000
40ACN	100	200	67	200	40	1000
50ACN	100	250	71	150	50	1000
60ACN	100	300	75	100	60	1000
70ACN	100	350	78	50	70	1000
75ACN	100	375	79	25	75	1000

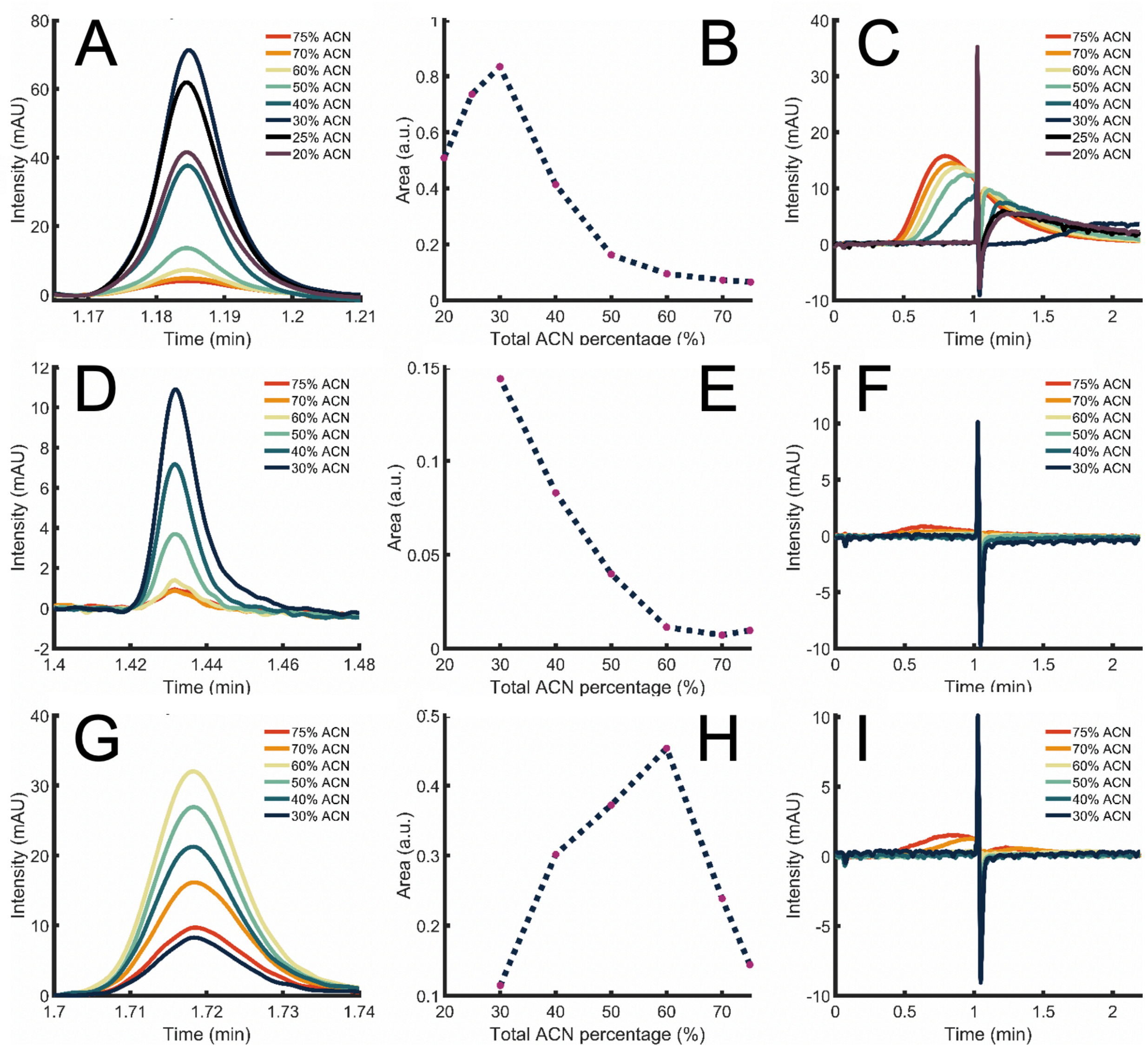

Figure S10. Results of the online NP disassembly experiments using the 2D-LC system for (A-C) curcumin, (D-F), coumarin-6, and (G-I) Sudan-IV. (A, D, G) Traces recorded by the elution detector, and (C, F, I) by the waste detector; (B, E, H) Peak areas were observed for the respective dyes using the elution detector.

S-VIII Optimized HDC × RPLC analysis

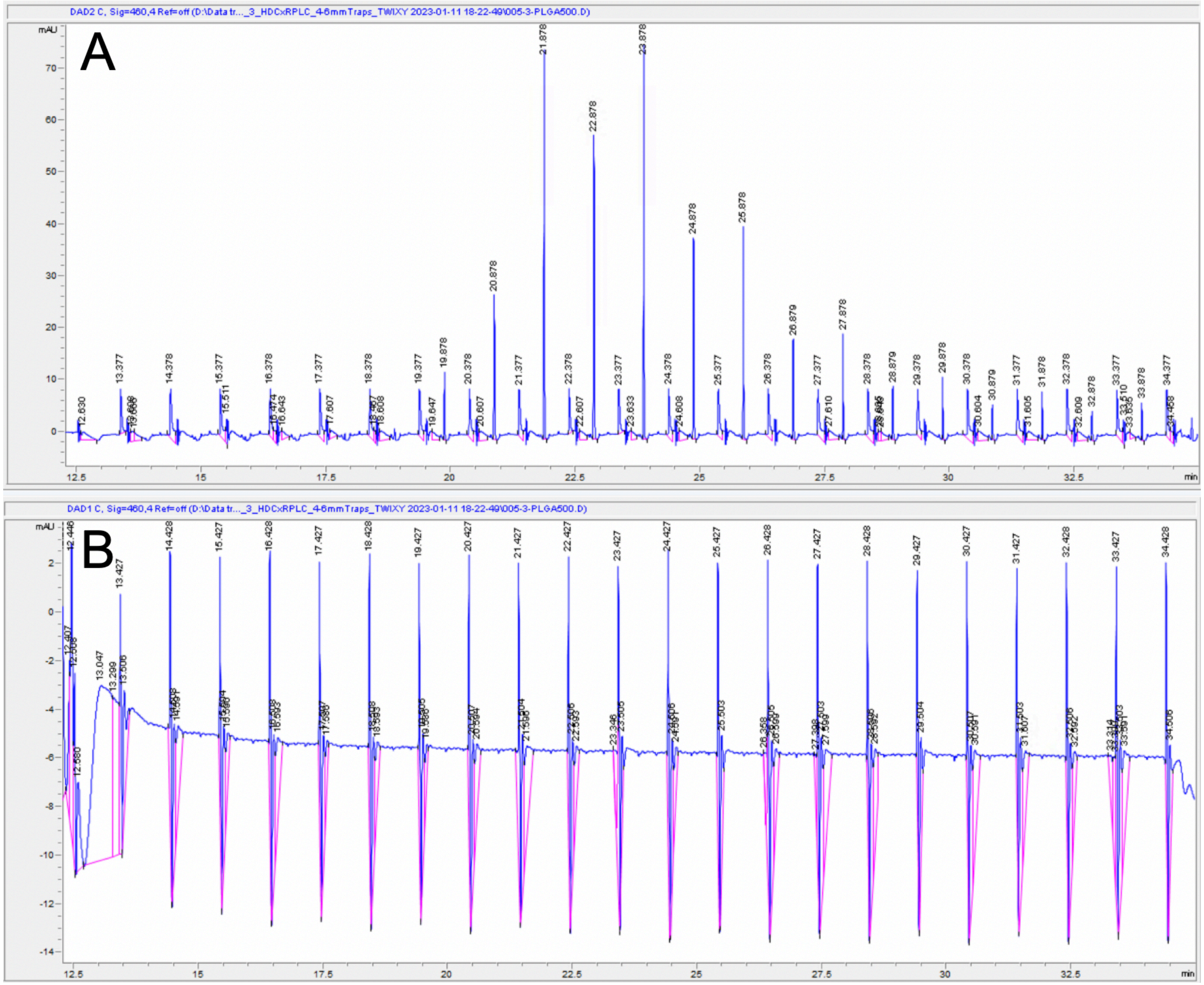

Figure S11. ^{2}D modulations of the HDC × RPLC separation of PLGA NP A. (A) signal from the elution detector; (B) signal from the waste detector.

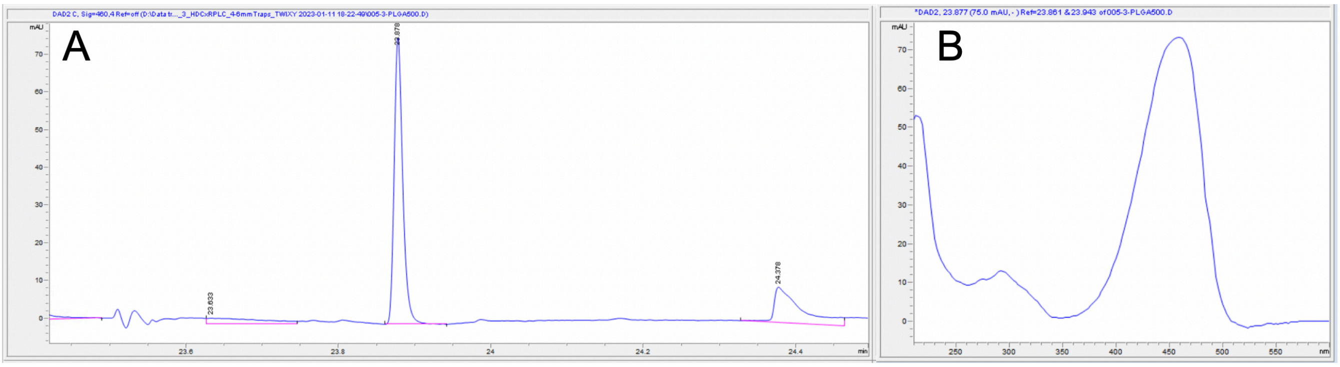

Figure S12. (A) Zoom-in of the most intense ^{2}D modulation of the HDC × RPLC separation of PLGA NP A; (B) corresponding UV spectrum.

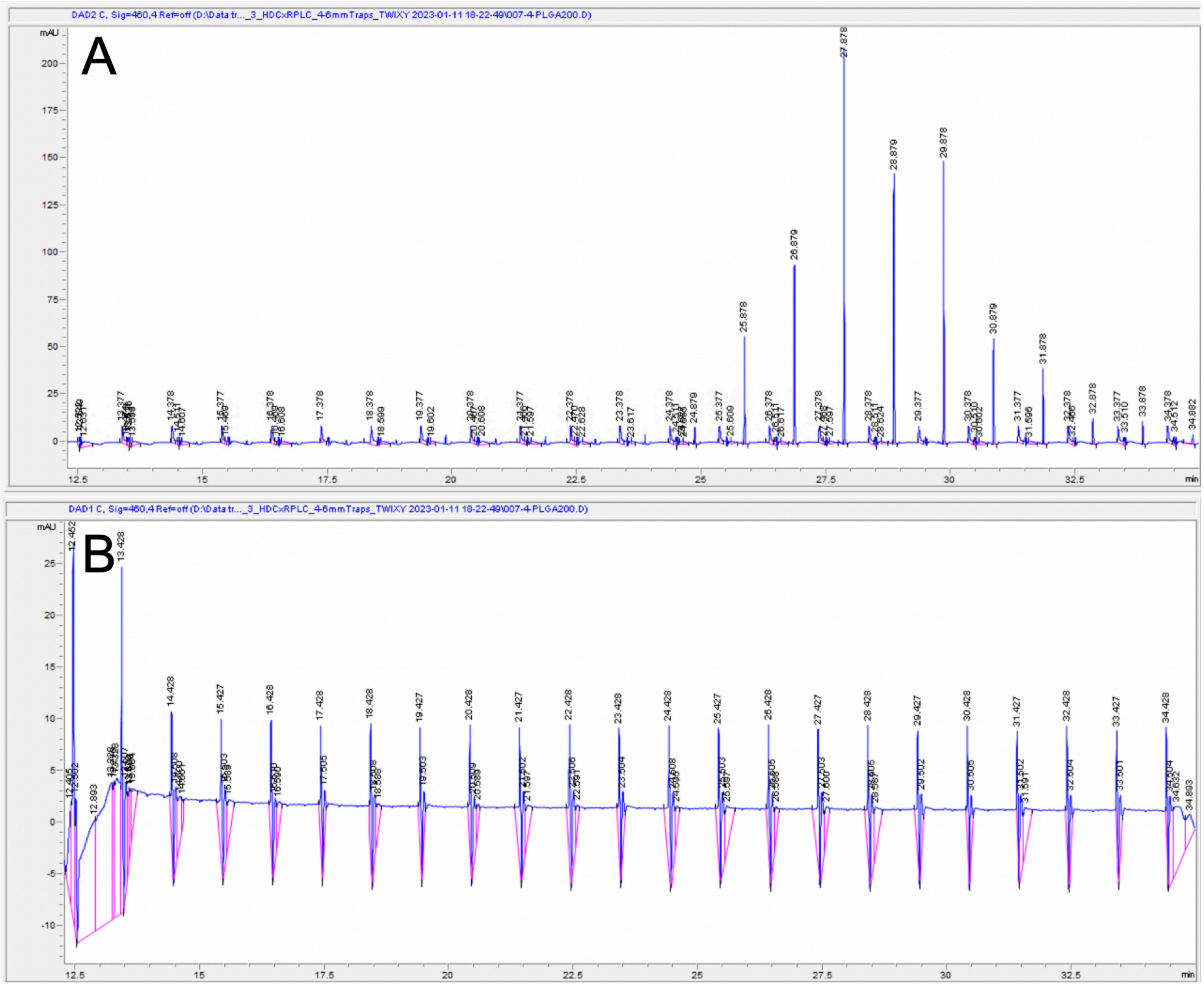

Figure S13. ^{2}D modulations of the HDC × RPLC separation of PLGA NP B. (A) signal from the elution detector; (B) signal from the waste detector.

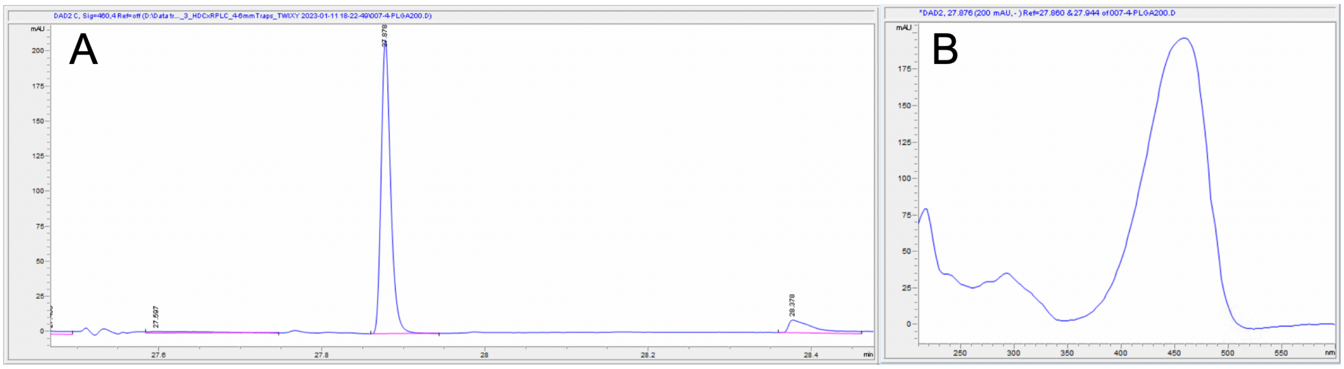

Figure S14. (A) Zoom-in of the most intense ^{2}D modulation of the HDC × RPLC separation of PLGA NP B; (B) corresponding UV spectrum.

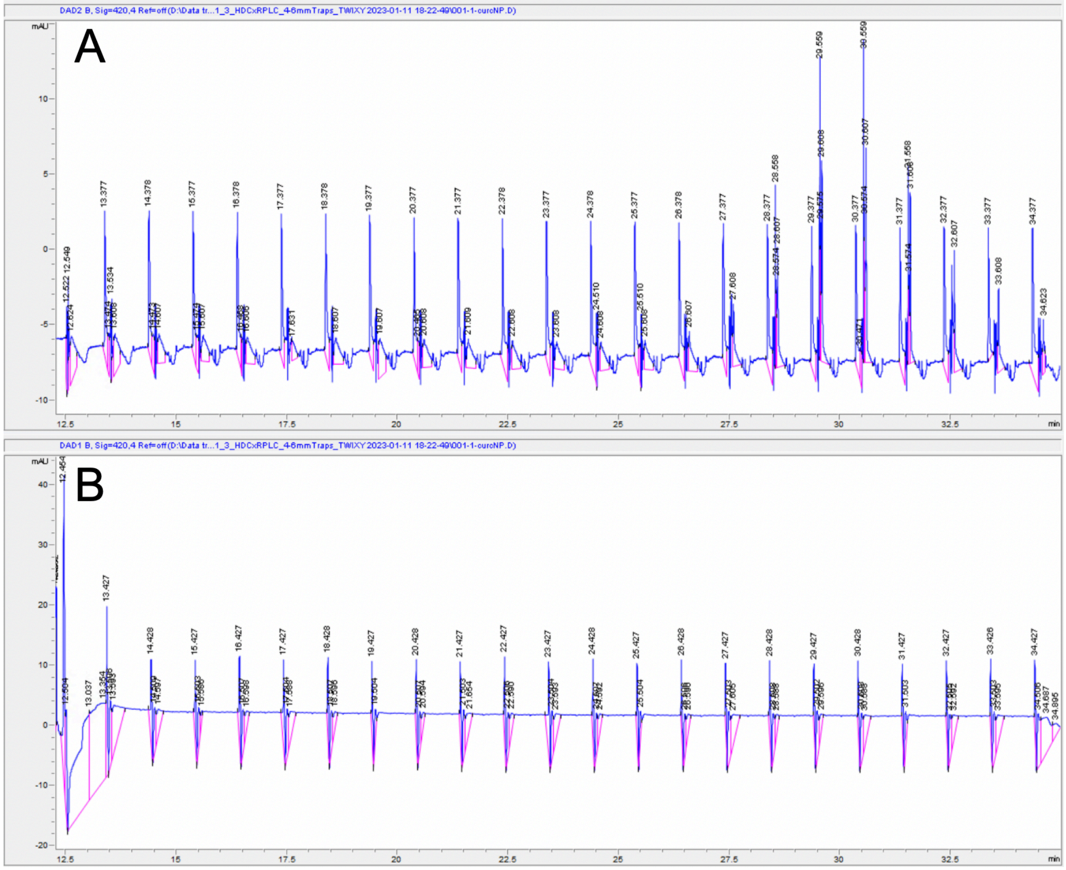

Figure S15 ^{2}D modulations of the HDC × RPLC separation of the curcumin NP. (A) signal from the elution detector; (B) signal from the waste detector.

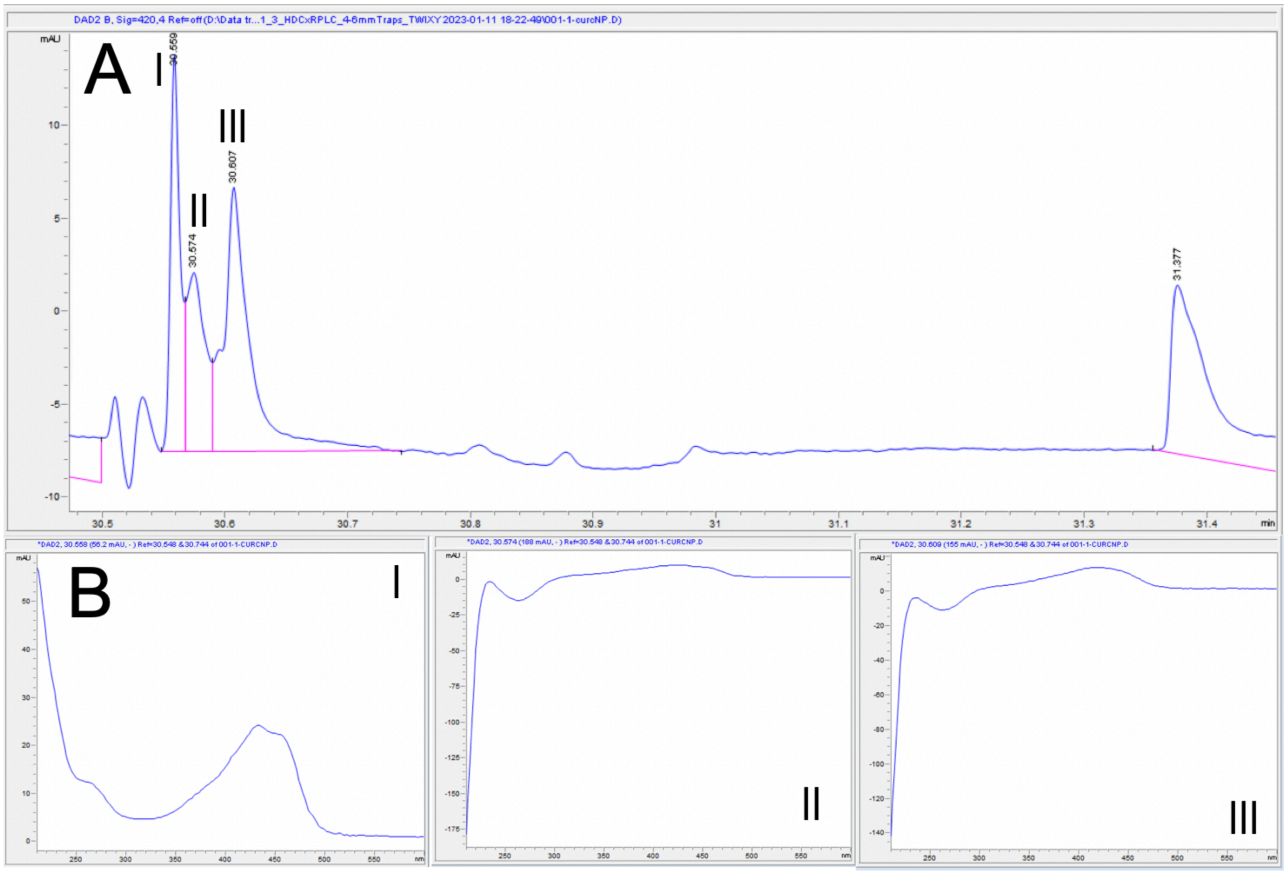

Figure S16. (A) Zoom-in of the most intense ^{2}D modulation of the HDC × RPLC separation of the curcumin NP; (B) corresponding UV spectrum.

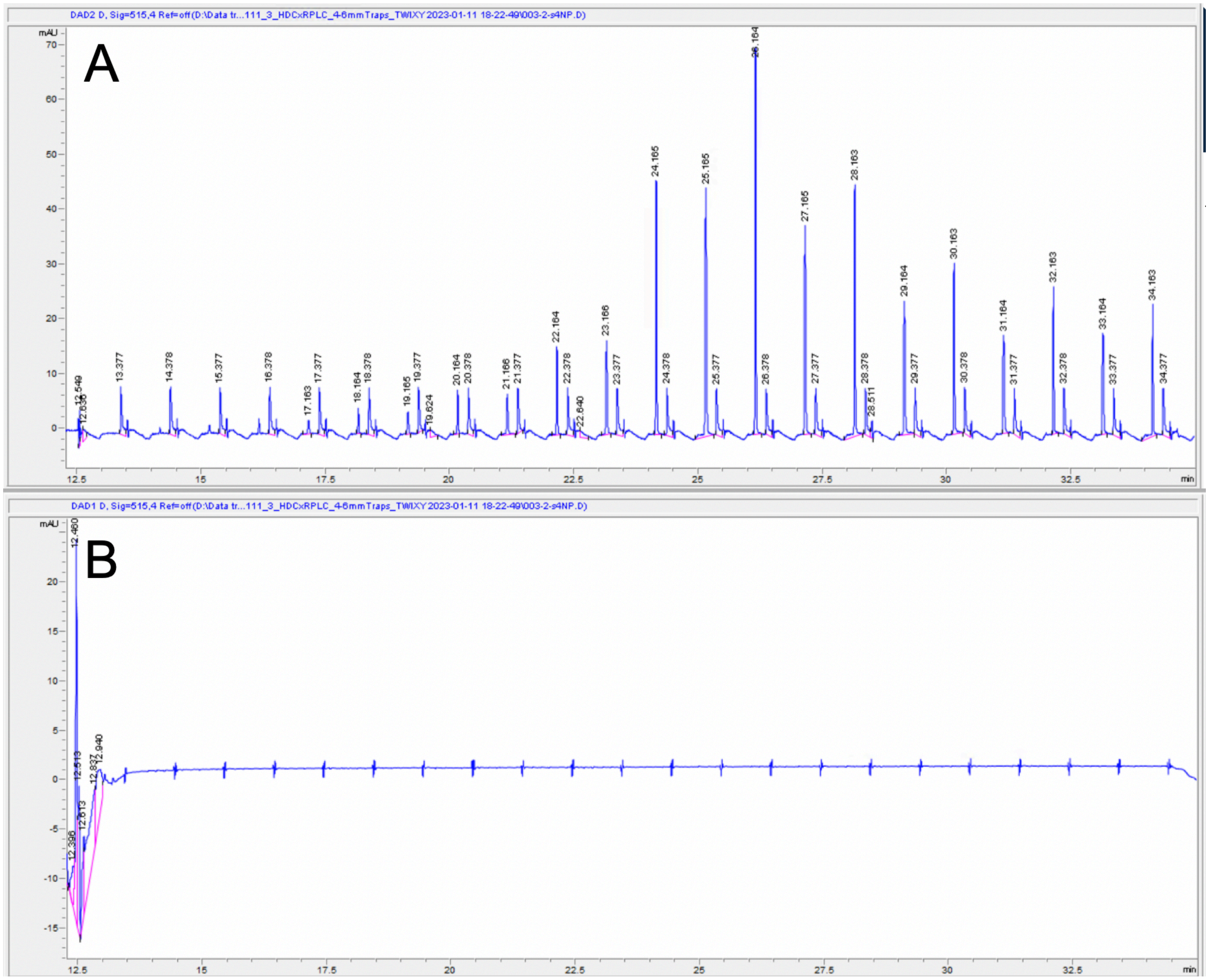

Figure S17. ²D modulations of the HDC × RPLC separation of the Sudan-IV NP. (A) signal from the elution detector; (B) signal from the waste detector.

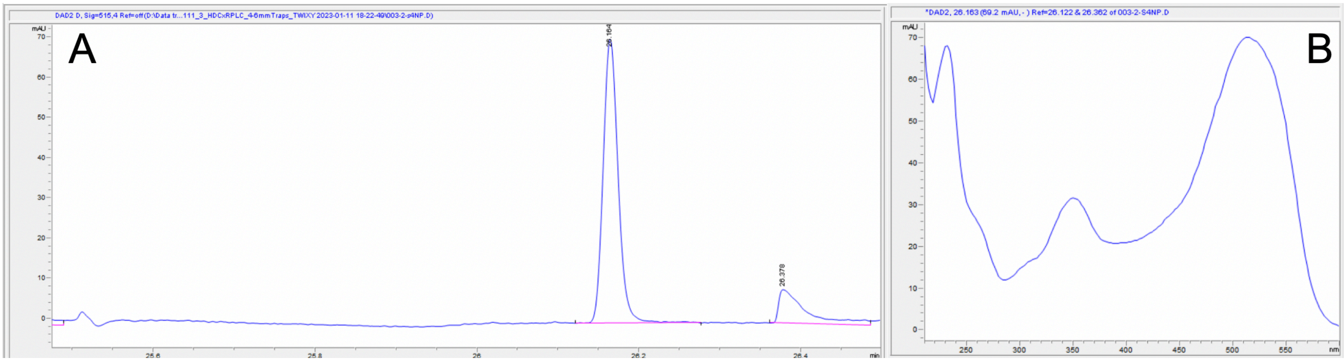

Figure S18. (A) Zoom-in of the most intense ²D modulation of the HDC × RPLC separation of the Sudan-IV NP; (B) corresponding UV spectrum.

For the calculation of the areas per modulation, only values above the LOQ are reported (Table S4). All 2D-LC runs were measured in 3 technical replicates. The trap positions alternated per modulation between 1 and 2, with each 2D-LC run starting at position 1. The calibration line for the specific trap position was applied to calculate the peak area per modulation. The total number of modulations considered for integration was 21. The total concentration of encapsulated dye was calculated by taking the sum of the concentrations per modulations divided by the V_{inj} (100 μL for PLGA NP A, 50 μL for the other NP samples).

Table S7: Mean dye concentrations, standard deviation (std) and relative standard deviation (rsd) values for the four NP samples that were measured with the optimized HDC × RPLC runs (n=3).

NP sample	Dye concentration in NP (mg L^{-1})	std (mg L^{-1})	rsd (%)
PLGA NP A	1.15	0.11	10%
PLGA NP B	0.96	0.01	1%
Curcumin NP	0.27	0.054	20%
Sudan-IV NP	1.99	0.031	2%

S-IX Extended HDC × RPLC separation of curcumin-loaded PLGA-PEG-PLGA NP

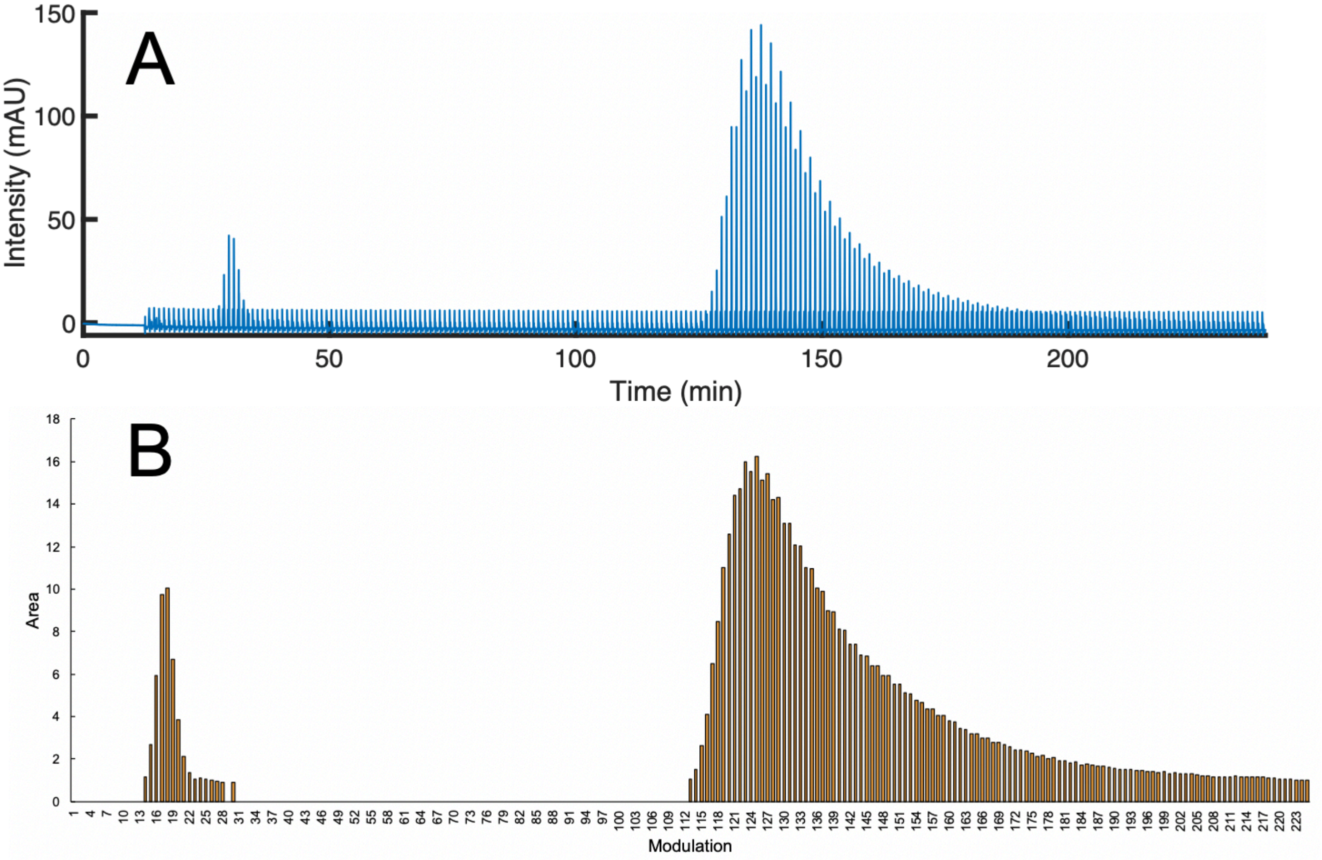

Figure S19. Extended HDC × RPLC separation of curcumin-loaded PLGA-PEG-PLGA NP. (A) raw ^{2}D RPLC chromatograms; (B) peak area of curcumin per modulation. The run consisted of 225 modulations. The left peak represents the actual HDC distribution of curcumin-loaded NPs and the right peak represents the retained non-encapsulated curcumin. The total concentration of all modulations was quantified to be 5.79 mg L^{-1}.
